# Nuclear blebs are composed of variable chromatin states and display increased transcription initiation relative to elongation

**DOI:** 10.1242/bio.062585

**Published:** 2026-10-30

**Authors:** Madeleine Clark, Antonela Losada, Sonia Jahng, Allen Saini, Fawwaz Chowhan, Gwyneth Woods, Adelaide Cutler, Stephanie Hallerman, Minnah Gayed, Sayali Bhalerao, Emanuel Bullock, Chris Santry, Adriana Panagiotou, Bailey Lapolla, Nitish Bhatta, Savanah Freidus, Gurnoor Kaur, David Bai, Daniel Hu, Kian Tadbiri, Mary Packard, Katherine Dorfman, Nickolas Borowski, Kelsey Prince, Nick Lang, Carline Fermino Do Rosario, Andrew D. Stephens

**Affiliations:** ^1^ Biology Department, University of Massachusetts Amherst, Amherst, MA 01003, USA; ^2^ Molecular and Cellular Biology, University of Massachusetts Amherst, Amherst, MA 01003, USA

**Keywords:** Nucleus, Nuclear bleb, Histones, Euchromatin, Heterochromatin, Transcription initiation

## Abstract

Nuclear blebs are herniations of the nucleus that occur in many human conditions including aging, heart disease, muscular dystrophy, and many cancers. Nuclear blebbing causes nuclear rupture and cellular dysfunction. However, understanding the formation, stability, and identification of nuclear blebs remains an ongoing challenge. Our previous studies revealed that nuclear blebs are best hallmarked by decreased DNA density. To determine if chromatin decompaction underlies decreased DNA density in nuclear blebs, we investigated the histone composition of nuclear blebs across multiple cell lines. Time-lapse and immunofluorescence imaging revealed that histone levels decreased in the nuclear bleb compared to the nuclear body but were more variable than DNA. Next, we imaged histone modification states of euchromatin and heterochromatin, which track the decondensed and compact states of chromatin, respectively. We found that nuclear blebs displayed variable histone modification states across cell lines, as euchromatin is not consistently enriched nor is heterochromatin consistently depleted. Nuclear blebs did consistently show that active RNA polymerase II initiation is significantly increased relative to elongation. Thus, decreased DNA density that hallmarks nuclear blebs is not due to local histone modification state, and the increase in transcription initiation relative to elongation is reproducible in nuclear blebs.

## Teaser

**Summary:** We measured histones and their modification states in nuclear blebs. We found that the chromatin state is variable while transcription initiation is consistently enriched relative to elongation in nuclear blebs.

## INTRODUCTION

The nucleus is the organelle that houses the genome and its essential functions. Abnormal nuclear morphology is a hallmark of human afflictions including advanced aging, heart disease, muscular dystrophy, and many cancers (Stephens et al., 2019a; Kalukula et al., 2022). A specific type of abnormal nuclear morphology is a nuclear bleb that presents as a herniation of the nucleus with decreased DNA density (Bunner et al., 2025; Pujadas Liwag et al., 2025) and is common in progeria, an advanced aging disease, and in many cancers (Helfand et al., 2012; Stephens et al., 2019a). Many factors that affect nuclear blebbing levels have been reported, including nuclear strength via chromatin compaction and lamin levels (Lammerding et al., 2006; Furusawa et al., 2015; Denais et al., 2016; Stephens et al., 2018; 2019b; Kidiyoor et al., 2020; Strom et al., 2021; Pho et al., 2024), actin confinement and contraction (Le Berre et al., 2012; Hatch and Hetzer, 2016; Pho et al., 2023), and transcriptional activity (Helfand et al., 2012; Berg et al., 2023; Prince et al., 2026). However, the process by which nuclear blebs form and stabilize remains to be determined.

We reasoned that the composition of the nuclear bleb would provide insights into how nuclear blebs form, as suggested in a previous review (Srivastava et al., 2021). Our recent work revealed that decreased DNA density is a universal marker of nuclear blebs whereas lamin B is only lost upon nuclear bleb rupture (Bunner et al., 2025; Chu et al., 2025). One hypothesis for decreased DNA density in nuclear blebs is that decondensed euchromatin, marked by histone acetylation H3K27ac and H3K9ac, enriches nuclear blebs as previously reported (Bercht Pfleghaar et al., 2015; Stephens et al., 2018). This hypothesis includes the prediction that compact heterochromatin marked by histone methylation at H3K27me3 and H3K9me2,3 would be depleted in nuclear blebs. However, comprehensive quantification of nuclear bleb chromatin state across cell types and conditions remains deficient.

An alternative hypothesis is that transcriptional activity is essential for the formation and stabilization of nuclear blebs locally. Transcriptional activity has been shown to be important for nuclear bleb formation and stability (Berg et al., 2023; Prince et al., 2026). Transcriptional activity can be inferred by measurement of the phosphorylation state of RNA polymerase II (RNA Pol II), in which phosphorylated Serine 5 and Serine 2 residues denote transcription initiation and elongation, respectively (Komarnitsky et al., 2000; Schwer and Shuman, 2011).

To determine the composition of nuclear blebs within the context of histones and histone modification states, we performed immunofluorescence and measured the fluorescence signal in the nuclear bleb relative to the nuclear body across multiple cell lines. First, we imaged global histone H2B and H3 levels via fluorescently tagged proteins and immunofluorescence, respectively. Next, we measured nuclear bleb to body ratios for euchromatin histone modifications H3K9ac and H3K27ac and heterochromatin histone modifications H3K9me2,3 and H3K27me3. Finally, we assayed levels of active RNA Pol II via pSer2/5 fluorescence intensities, which provide transcription elongation and initiation, respectively. Overall, we found that chromatin histone modification state varies across cell lines and conditions, while transcriptional initiation activity is consistently enriched in nuclear blebs relative to transcription elongation.

## RESULTS

### Histones are depleted in the nuclear bleb relative to the nuclear body

Decreased DNA density has been shown to be the best and most reproducible hallmark for nuclear blebs. We hypothesized that histones would show a similar consistent decrease in the nuclear bleb. A nuclear localization signal (NLS-GFP) was used as a control to determine how a diffusible fluorescence protein would report the nuclear bleb-to-body intensity. To measure both simultaneously, we imaged histone H2B-mCherry and NLS-GFP in stably expressing HT1080 cell lines. All measurements of the nuclear bleb fluorescence intensity are made relative to the nuclear body, providing a nuclear bleb-to-body ratio. As previously reported, the vast majority of nuclear blebs rupture frequently, which results in spilling of nuclear contents that could impact bleb-to-body measurements. Thus, measurements were taken at least 4 min before a nuclear rupture. For nuclei in between nuclear ruptures, we found that NLS-GFP intensity is slightly but significantly decreased in the nuclear bleb relative to the nuclear body (*P*<0.05, Fig. S1A) and has a nuclear bleb-to-body ratio of 0.86±0.01 (mean±s.e.m., Fig. 1A). The H2B-mCherry bleb-to-body ratio has a statistically significant decrease of 0.58±0.01 relative to that of NLS-GFP (Fig. 1A). These data reveal that histone H2B has significantly decreased density in the nuclear bleb-to-body ratio compared to a diffusible NLS-GFP.

**Fig. 1.**
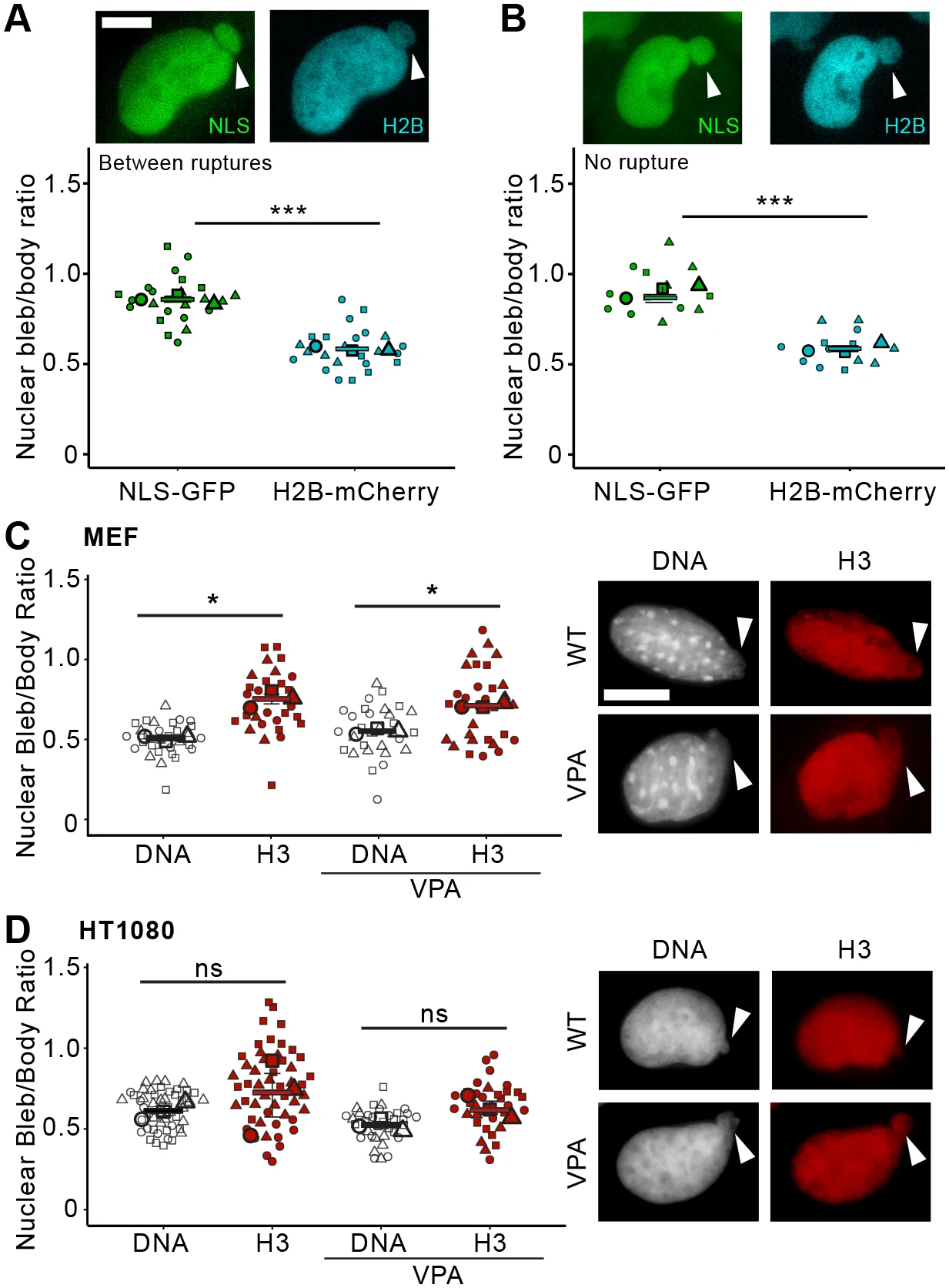
**Histone nuclear bleb-to-body ratios are less than that of NLS-GFP but higher relative to that of DNA.** (A,B) Example images of a nuclear bleb in HT1080 cells stably expressing NLS-GFP (green) and H2B-mCherry (blue). Super plots of nuclear bleb-to-body ratios (bleb/body) of NLS-GFP and H2B-mCherry (A) in cells in between nuclear ruptures (biological triplicates *n*=7, 7, 9) and (B) in cells with no nuclear rupture taken every hour (biological triplicates *n*=5, 5, 3). (C,D) Example images and super plots of nuclear bleb-to-body ratios for the Hoechst 33342 DNA stain (gray) and H3 (red) for cell lines and drug treatments, biological triplicates: (C) MEF WT *n*=8, 15, 7 and MEF VPA *n*=13, 11, 10 and (D) HT1080 WT *n*=10, 20, 20 and HT1080 VPA *n*=8, 9, 13. White arrowheads denote the nuclear bleb. Mean±s.e.m. is graphed. Statistical significance is denoted by *P<0.05, ***P<0.001 or ns (not significant) via two-tailed paired Student's *t*-test. Scale bars: 10 μm.

Time-lapse imaging of rare non-rupturing nuclear blebs was done to determine if the decrease in H2B relative to NLS-GFP in the nuclear bleb was dependent on nuclear rupture. Three HT1080 nuclei that had stable non-rupturing nuclear blebs were measured every hour for the bleb-to-body ratios of both NLS-GFP and H2B-mCherry. These non-rupturing blebs had bleb-to-body ratios similar to those of rupturing blebs (Fig. S1B). We found that non-rupturing nuclear blebs have a significantly decreased H2B nuclear bleb-to-body ratio of 0.58±0.02 relative to the 0.91±0.02 ratio of NLS-GFP (Fig. 1B). Thus, imaging of tagged fluorescence cell lines confirms that histone levels are significantly decreased in the nuclear bleb relative to the nuclear body, independent of nuclear rupture.

We then conducted immunofluorescence and fluorescence imaging experiments to compare the bleb-to-body ratios of DNA to those of histone H3. Wild-type (WT) mouse embryonic fibroblasts (MEFs) were fixed, stained for DNA imaging via Hoechst 33342, and labeled for histone H3 via an antibody. Imaging and analysis of nuclear bleb-to-body ratios recapitulated decreased DNA density to ≤0.56±0.03, in agreement with our previous publications (Bunner et al., 2025; Chu et al., 2025). Histone H3 immunofluorescence measured a nuclear bleb-to-body ratio of 0.75±0.03 in WT MEFs and 0.72±0.01 in MEFs treated with histone deacetylase inhibitor valproic acid (VPA), which increases euchromatin and nuclear blebbing (Fig. 1C). For both WT and VPA-treated MEFs, the nuclear bleb-to-body ratio of H3 relative to DNA increased. Nuclear blebs in the human cell line HT1080 had H3 nuclear bleb-to-body ratios of 0.69±0.16 for WT MEFs and 0.63±0.04 for VPA-treated MEFs (Fig. 1D). The HT1080 DNA nuclear bleb-to-body ratio was statistically similar to that of H3. Furthermore, we repeated these experiments in prostate cancer cell lines LNCaP, PC3, and DU145. Prostate cancer cell lines all showed statistically similar H3 and DNA nuclear bleb-to-body ratios of 0.5-0.6 (Fig. S1). Overall, global levels of histones H2B and H3 decrease in the nuclear bleb relative to the body, though they show greater variability than decreased DNA density.

### Nuclear blebs are not consistently enriched in euchromatin

Since nuclear blebs consistently have decreased DNA density, we hypothesized that they would also have decondensed chromatin. To measure levels of euchromatin, which is the decondensed chromatin state, we assayed the nuclear bleb-to-body ratios of the immunofluorescence markers H3K27ac and H3K9ac along with the DNA stain Hoechst 33342 in MEFs (Fig. 2A). In WT MEFs, we found that both H3K27ac and H3K9ac exhibited variable bleb-to-body ratios that did not show a statistically significant difference from those of DNA.

**Fig. 2.**
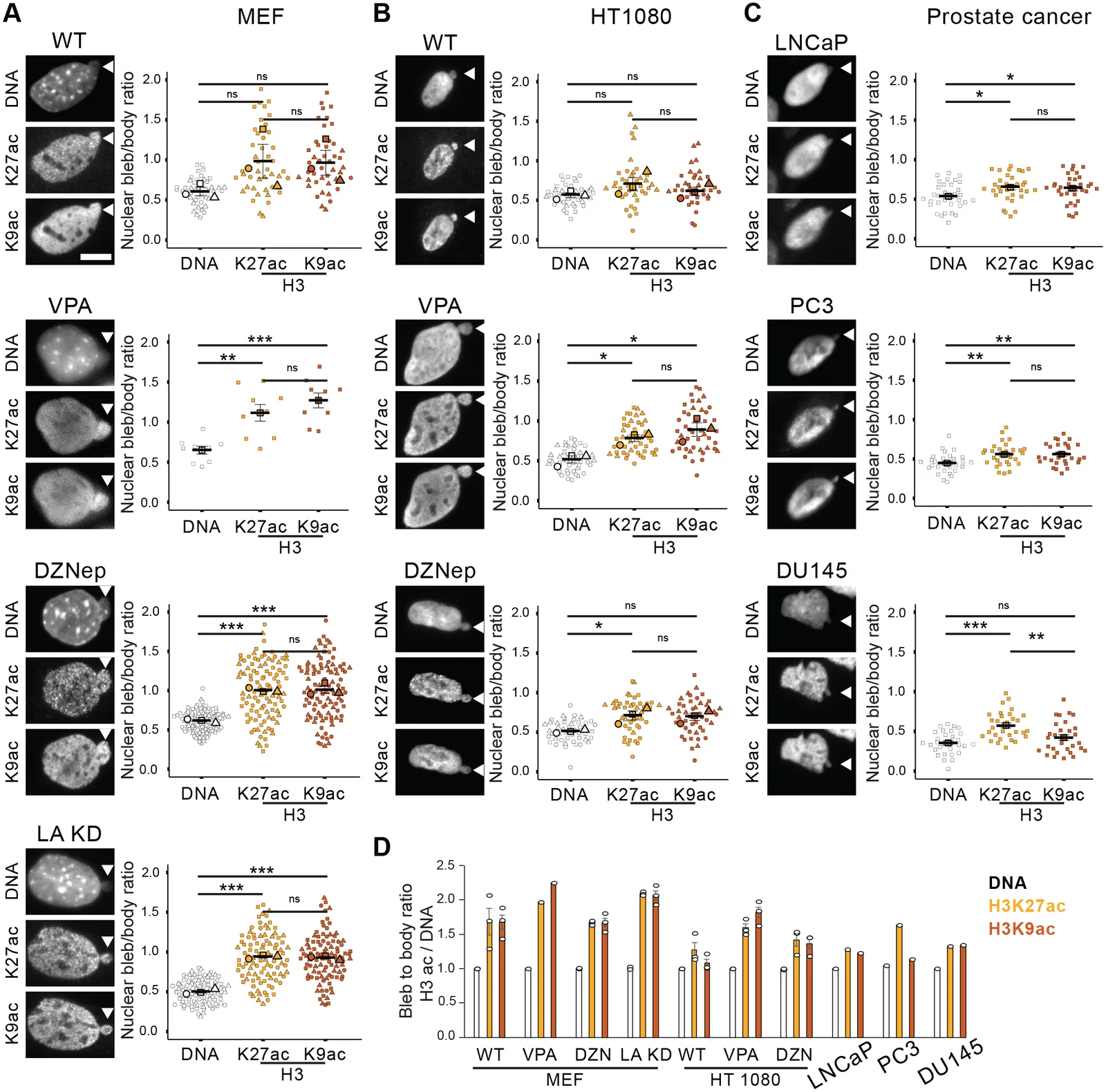
**Euchromatin immunofluorescence shows variable nuclear bleb-to-body ratios across cell lines and conditions.** (A-C) Examples of nuclear blebs in (A) MEF cells, (B) human HT1080 cells, and (C) human prostate cancer cell lines imaged via Hoechst (DNA) and euchromatin markers H3K27ac and H3K9ac. White arrowheads denote the nuclear bleb. Super plots of nuclear bleb-to-body ratios from biological triplicates in (A) MEF and (B) HT1080 WT, showing increased euchromatin (VPA), decreased heterochromatin (DZNep), and lamin A knockdown (LA KD, MEF only). (C) Super plots of nuclear bleb-to-body ratios in human prostate cancer cell lines LNCaP (*n*=33), PC3 (*n*=30), and DU145 (*n*=29). All data were collected as biological triplicates, except MEF VPA and prostate cancer cell lines. (D) Summary graph of all cell lines and conditions for bleb-to-body H3 acetylation (ac) markers divided by DNA for biological averages. Statistics are the same as original data graphs in panels A-C. Mean±s.e.m. is graphed in A, B, and C. Statistical significance is denoted by *P<0.05, **P<0.01, ***P<0.001 or ns (not significant) via one-way ANOVA with post hoc Tukey test. Scale bar: 10 μm.

Nuclear blebbing is known to increase upon perturbation of chromatin compaction via inhibition of histone modification enzymes (Stephens et al., 2018; Kalinin et al., 2021; Manning et al., 2025). DNA and euchromatin markers in nuclear blebs were then assayed in cells treated with histone deacetylase inhibitor VPA, which increases euchromatin, or histone methyltransferase inhibitor 3-deazaneplanocin A (DZNep), which decreases heterochromatin. Euchromatin markers in these chromatin perturbations showed a significantly elevated nuclear bleb-to-body ratio compared to the decreased DNA density (Fig. 2A, VPA and DZNep). Nuclear blebbing can also be increased by lamin A knockdown (Vahabikashi et al., 2022; Berg et al., 2023; Pho et al., 2024). In lamin-A-knockdown MEFs, nuclear blebs again showed a consistent and typical loss of DNA density while euchromatin markers showed a relative increase in the nuclear bleb-to-body ratio (Fig. 2A, LA KD).

To determine if an increased euchromatin bleb-to-body ratio compared to DNA is consistent, we measured immunofluorescence in a second cell type, human fibrosarcoma HT1080 cells. HT1080 WT and chromatin decompaction conditions achieved through VPA or DZNep treatment revealed variable behaviors for euchromatin markers (Fig. 2B). WT again showed that no euchromatin marker increased in nuclear bleb-to-body ratio relative to that of DNA. In VPA both euchromatin markers increased nuclear bleb-to-body ratio relative to that of DNA. In DZNep, the nuclear bleb-to-body ratio of H3K27ac increased relative to that of DNA, but not H3K9ac. Thus, euchromatin is not broadly enriched relative to DNA in the nuclear bleb across cell lines or conditions.

We used three human prostate cancer cell lines that provide different nuclear bleb rupture incidence rates. Blebbed nuclei in PC3 cells rupture frequently, similarly to MEFs and HT1080 cells, whereas LNCaP and DU145 rarely rupture (Chu et al., 2025). Similar to other cell lines, enrichment of euchromatin markers relative to DNA in the bleb was not consistent. Specifically, the H3K9ac nuclear bleb-to-body ratio in DU145s was not increased relative to DNA (bleb-to-body ratio of <0.5, Fig. 2C). Overall, the data show that euchromatin, the decondensed chromatin state, is not enriched in the nuclear bleb relative to the nuclear body, as most nuclear bleb-to-body average ratios are not greater than 1. Furthermore, euchromatin is not consistently higher than DNA density in the nuclear bleb relative to the body.

### Constitutive heterochromatin shows a differential behavior in nuclear bleb-to-body ratio relative to DNA

A decrease in chromatin density in the nuclear bleb could result from a loss of heterochromatin and the associated compact chromatin state. To determine the presence of heterochromatin in nuclear blebs, we assayed the facultative heterochromatin marker H3K27me3 and the constitutive heterochromatin marker H3K9me2,3 (Lee et al., 2020). In WT MEFs, blebs are largely devoid of facultative heterochromatin marker H3K27me3, which shows a decreased bleb-to-body ratio of 0.8±0.02 (Fig. 3A). In contrast, the constitutive heterochromatin marker H3K9me2,3 is maintained with a nuclear bleb-to-body ratio of 1.0±0.12, which is significantly higher than that of DNA.

**Fig. 3.**
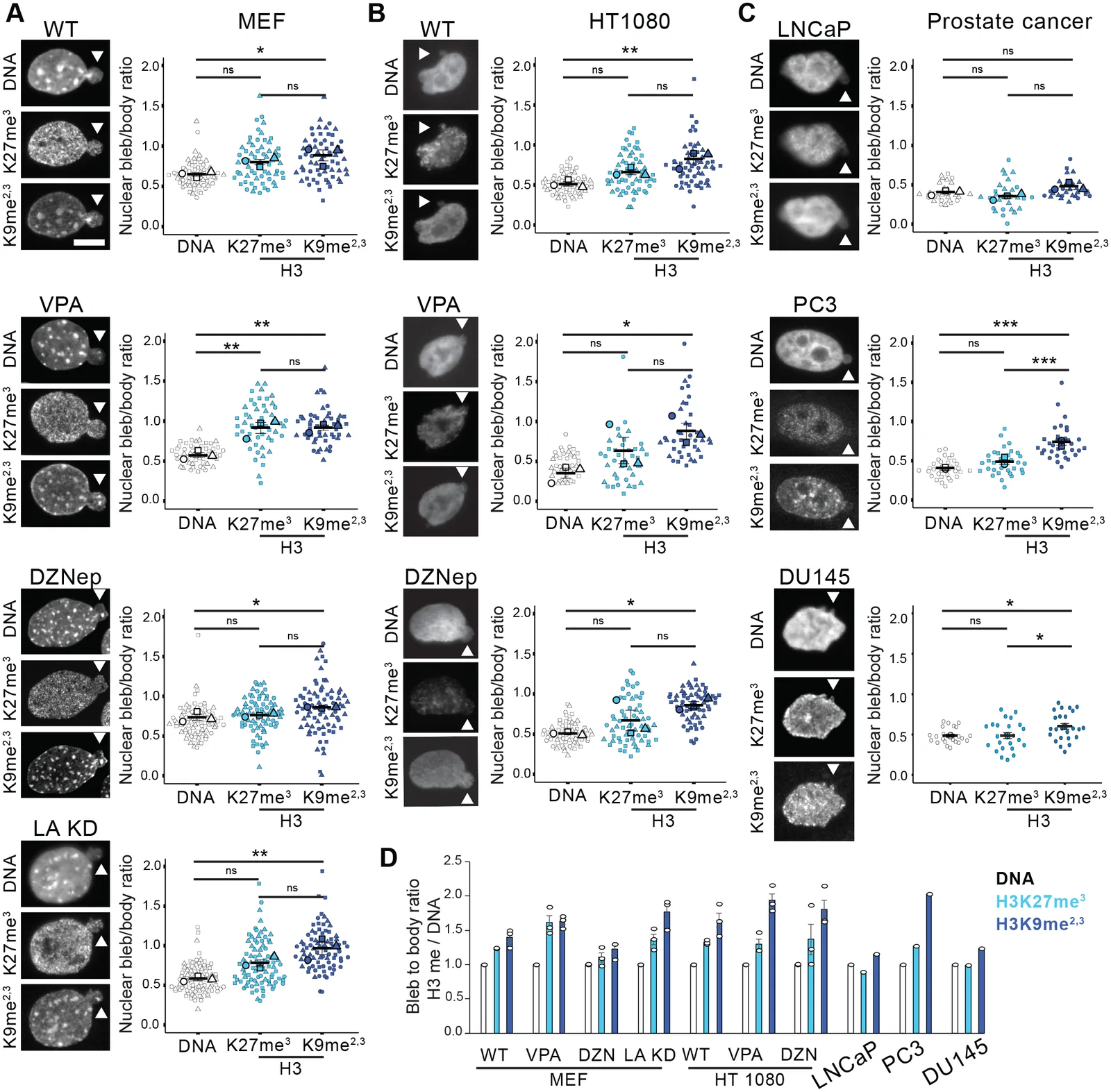
**Facultative and constitutive heterochromatins show differential nuclear bleb-to-body ratios relative to those of DNA.** (A-C) Examples of nuclear blebs in (A) MEF cells, (B) human HT1080 cells, and (C) human prostate cancer cell lines imaged via Hoechst (DNA) and heterochromatin markers H3K27me3 and H3K9me2,3. White arrowheads denote the nuclear bleb. Super plots of nuclear bleb-to-body ratios from biological triplicates in (A) MEF and (B) HT1080 WT, showing increased euchromatin (VPA), decreased heterochromatin (DZNep), and lamin A knockdown (LA KD, MEF only). (C) Super plots of nuclear bleb-to-body ratios in human prostate cancer cell lines LNCaP (*n*=10, 10, 9), PC3 (*n*=11, 11, 11), and DU145 (*n*=24). All data were collected as biological triplicates, except PC3 and DU145. (D) Summary graph of all cell lines and conditions for bleb-to-body H3 methylation (me) markers divided by DNA for biological averages. Statistics are the same as original data graphs in panels A-C. Mean±s.e.m. is graphed in A, B, and C. Statistical significance is denoted by *P<0.05, **P<0.01, ***P<0.001 or ns (not significant) via one-way ANOVA with post hoc Tukey test. Scale bar: 10 μm.

Next, we assayed nuclear blebs under conditions that increase nuclear blebbing. For MEFs, we found that the nuclear bleb-to-body ratios of facultative heterochromatin are similar to those of DNA in most, but not all, conditions (Fig. 3A, H3K27me3 in WT, DZNep, and LA KD). Interestingly, constitutive heterochromatin in all MEF conditions measures a statistically significant increase in the bleb-to-body ratio relative to that of DNA (Fig. 3A, H3K9me2,3). Thus, in MEF cells, the nuclear bleb-to-body ratio of facultative heterochromatin is similar to that of DNA, while the ratio of constitutive heterochromatin is increased relative to DNA.

To determine the consistency of nuclear bleb heterochromatin composition, we analyzed human fibrosarcoma HT1080 WT and chromatin compaction perturbation conditions. Facultative heterochromatin H3K27me3 showed a decreased bleb-to-body ratio similar to that of DNA (Fig. 3B). Similar to MEFs, HT1080 constitutive heterochromatin H3K9me2,3, relative to DNA, had increased nuclear bleb-to-body ratios. Taken together, constitutive heterochromatin is not depleted in the nuclear bleb relative to the nuclear body like DNA while facultative heterochromatin is depleted, similar to DNA.

Prostate cancer cell lines, which have different rupture frequencies, further test the consistency of nuclear bleb composition. Again, prostate cancer cell lines had a spectrum of slightly different outcomes. LNCaP cells reveal similar decreased nuclear bleb-to-body ratios across DNA and facultative and constitutive heterochromatin (Fig. 3C, LNCaP). In contrast, PC3 and DU145 appear similar to MEF and HT1080. In PC3 and DU145, the decreased facultative heterochromatin H3K27me3 bleb-to-body ratio is similar to that of DNA while the constitutive heterochromatin H3K9me2,3 bleb-to-body ratio is significantly increased compared to that of DNA (Fig. 3C, PC3 and DU145). Over multiple cell lines, facultative heterochromatin shows similarly depleted bleb-to-body ratios as DNA, while constitutive heterochromatin, relative to DNA, has an increased bleb-to-body ratio.

### Nuclear blebs show consistent enrichment of RNA Pol II transcription initiation versus elongation across cell lines

Since the histone modification state was variable across nuclear blebs, we aimed to determine a consistent component. Another major contributor to nuclear blebbing is transcriptional activity (Berg et al., 2023; Prince et al., 2026; Borowski et al., 2026 preprint). Transcriptional activity can be inferred through immunofluorescence measurements of active RNA Pol II phosphorylated at Serine 5 for initiation and Serine 2 for elongation (Komarnitsky et al., 2000; Barilla et al., 2001; Kim et al., 2004; Schwer and Shuman, 2011). In MEF nuclear blebs, RNA Pol II pSer5 (initiation) had a significantly higher nuclear bleb-to-body ratio than RNA Pol II pSer2 (elongation, Fig. 4A), in agreement with our previous publication (Berg et al., 2023). In the human cell line HT1080, we found a similar increased bleb-to-body ratio for RNA Pol II pSer5 relative to pSer2 (Fig. 4B). This same outcome was also found in VPA treatment of MEFs and HT1080 cells (Fig. S2). Thus, while histone modifications varied in the nuclear bleb-to-body ratio, RNA Pol II transcription initiation consistently increased compared to transcription elongation in nuclear blebs.

**Fig. 4.**
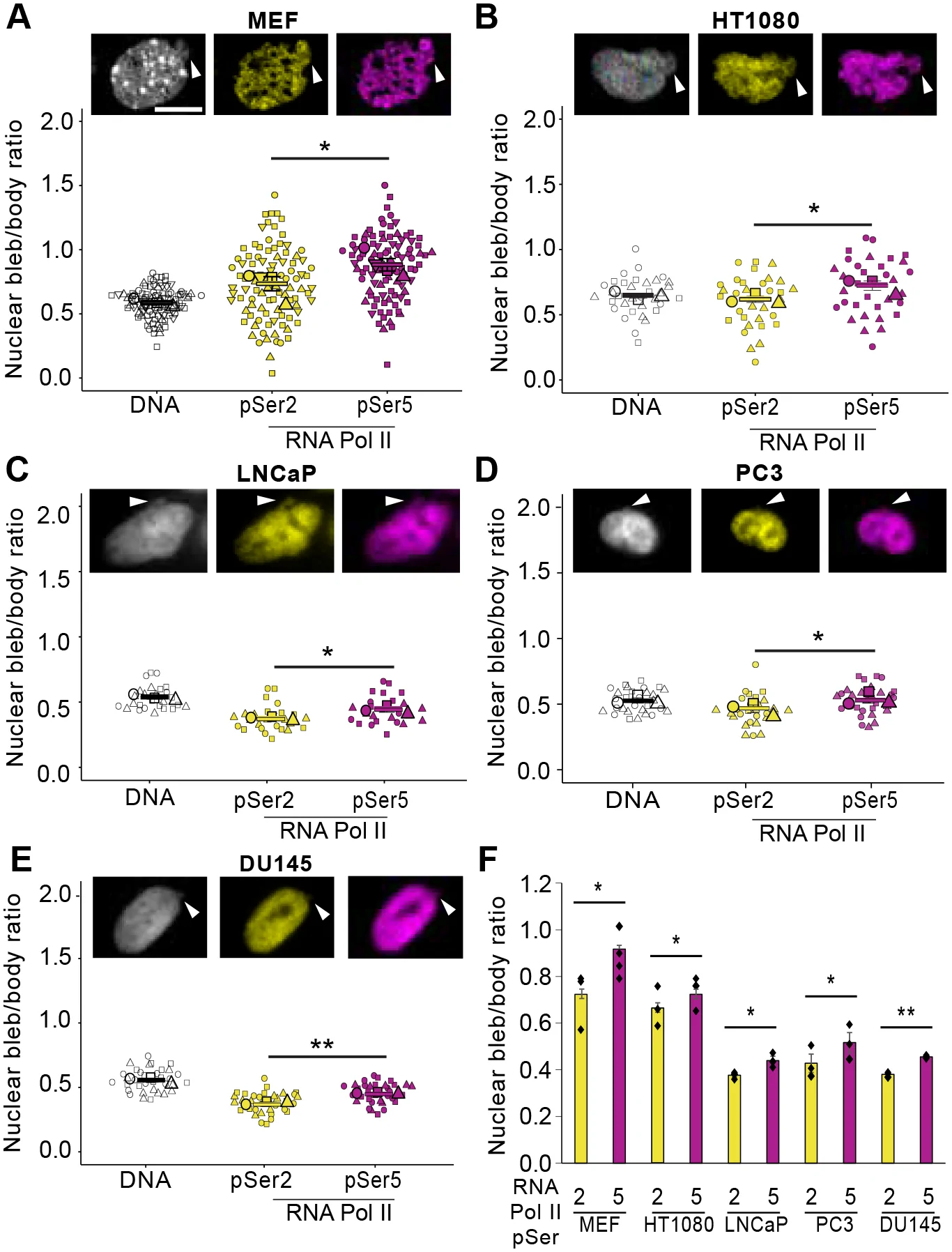
**Active RNA Pol II pSer5 initiation is enriched relative to pSer2 elongation in nuclear blebs.** (A-E) Example images and super plots of nuclear bleb-to-body ratio for DNA (gray) and RNA Pol II pSer2 (yellow, elongation) and pSer5 (magenta, initiation) for cell lines (A) MEF (*n*=18, 40, 20, 18), (B) HT1080 (*n*=10, 10, 12); (C) LNCaP (*n*=6, 10, 10), (D) PC3 (*n*=9, 7, 13), and (E) DU145 (*n*=12, 11, 11). All are biological triplicates or more. (F) The graph summarizing all cell lines shows a significant enrichment of RNA Pol II pSer5 relative to pSer2 in the nuclear bleb-to-body ratio. Mean±s.e.m. is graphed. Statistical significance between pSer2 and pSer5 is denoted by *P<0.05, **P<0.01 or ns (not significant) via two-tailed paired Student's *t*-test. Scale bar: 10 μm.

RNA Pol II pSer5 and pSer2 immunofluorescence in prostate cancer cells recapitulates findings in MEFs and HT1080 cells. LNCaP and DU145 cells rarely rupture, whereas PC3 cells rupture frequently, similar to MEFs and HT1080 cells (Chu et al., 2025). Across these cell lines with different rupture rates, transcription initiation remains higher in the bleb than transcription elongation (Fig. 4C-F). Conditions with chromatin state perturbations follow the same pattern, revealing that transcription initiation enrichment relative to elongation markers in the nuclear bleb is consistent regardless of chromatin state (Fig. S2) or relative to DNA (Fig. S3). All measured changes in the nuclear bleb-to-body ratio of RNA Pol II pSer5 relative to pSer2 are both small in percentage and statistically significant (Fig. 4F). Taken together, active RNA Pol II transcription initiation was consistently increased compared to transcription elongation in nuclear blebs across all measured cell lines in a small yet statistically significant manner.

## DISCUSSION

Global levels of histones decreased in the nuclear bleb relative to the nuclear body although not as consistently compared to the level of decreased DNA density in nuclear blebs (Bunner et al., 2025; Chu et al., 2025; Pujadas Liwag et al., 2025). In comparison with diffusible NLS-GFP, histones showed a significant decrease in the nuclear bleb relative to the body, revealing that the decrease is due to decreased density and not due to decreased volume. Time-lapse imaging of stable non-rupturing nuclear blebs confirms that rupture is not required for the decreased level of histones in the bleb-to-body ratio (Fig. 1B). Interestingly, while DNA and histone levels decrease in the nuclear bleb compared to the body, decreased DNA is a more consistent marker as histone H3 immunofluorescence levels showed more variability between conditions and cell lines. Overall, decreased DNA density remains the most reproducible hallmark of nuclear blebs.

### Decreased DNA density in nuclear blebs is not due to the histone modification state

The hypothesis that decreased DNA density in the nuclear bleb is due to euchromatin enrichment is not supported by our data. A few previous publications reported that euchromatin markers are enriched in nuclear blebs (Bercht Pfleghaar et al., 2015; Stephens et al., 2018). Our novel data across five cell lines and multiple conditions show that euchromatin enrichment in the nuclear bleb relative to the nuclear body is inconsistent. Our immunofluorescence data do not account for the histone modification state below the widefield resolution limit or transient chromatin states during nuclear bleb formation. An alternative hypothesis is that a forming nuclear bleb pulls in local chromatin independent of state. Previous studies have shown that increased euchromatin in the nucleus makes the nucleus less stiff (Shimamoto et al., 2017; Stephens et al., 2017; Hobson et al., 2020), causing greater levels of nuclear blebbing (Stephens et al., 2018; Kalinin et al., 2021). While global euchromatin levels have been shown to hinder nuclear mechanics and shape maintenance, local euchromatin enrichment in the nuclear bleb relative to the nuclear body is not required.

The lack of heterochromatin depletion in the nuclear bleb-to-body ratio further refutes our initial hypothesis that histone-modification-based chromatin compaction underlies a nuclear bleb. Taken together, the lack of euchromatin enrichment and heterochromatin depletion in nuclear blebs strongly refutes that decreased DNA density is caused by the histone modification state. Instead, these data support a hypothesis that local histone modifications are randomly drawn into a nuclear bleb, in agreement with the variable experimental outcomes. An alternative hypothesis to be tested in the future is that peripheral and highly crosslinked constitutive heterochromatin could be a driving force for nuclear blebs. This idea is supported by our data's agreement with physics simulations showing that peripheral heterochromatin enrichment might be required for nuclear blebbing (Attar et al., 2024). Meanwhile, facultative heterochromatin showed a consistently decreased nuclear bleb-to-body ratio similar to DNA, suggesting that the more interiorly located facultative heterochromatin is not typically pulled into the bleb upon formation. Data further supporting that histone modification levels are variable in nuclear blebs across cells and conditions come from other publications showing substantial levels of H3K27me3 in the nuclear bleb (Ivanov et al., 2013). The data suggest that nuclear blebs consistently presenting decreased DNA density is not directly due to H3K9 and H3K27 euchromatin enrichment and heterochromatin depletion.

It could be argued that the differences in etiology, or the cause, of the nuclear bleb could underlie the inconsistent nature of euchromatin and heterochromatin in nuclear blebs. However, an ever-mounting list of evidence suggests that all nuclear blebs share key characteristics. Nuclear blebs in WT and actin, chromatin or lamin perturbation conditions present with similar markers and rely on similar factors. Independent of condition, all nuclear blebs present with the hallmark of decreased DNA density (Stephens et al., 2018; Bunner et al., 2025; Chu et al., 2025; Pujadas Liwag et al., 2025). Furthermore, nuclear blebs are reliant on similar factors of force balance between actin confinement and contraction (De Vos et al., 2011; Le Berre et al., 2012; Hatch and Hetzer, 2016; Stephens et al., 2018; Mistriotis et al., 2019; Pho et al., 2023; Bunner et al., 2026) and nuclear mechanics (Stephens et al., 2018; Vahabikashi et al., 2022; Manning et al., 2025). Beyond this force balance and independent of condition, nuclear blebs rely on active transcription (Berg et al., 2023; Prince et al., 2026). We now propose that active RNA Pol II initiation (pSer5) is increased relative to elongation (pSer2) in the nuclear bleb as a novel consistent marker across cell lines and conditions (Fig. 4).

### Increased active RNA Pol II initiation relative to elongation is associated with nuclear blebs

Our data  contribute to a growing list of findings supporting that transcriptional activity is associated with nuclear blebbing. Transcriptional activity has been shown to drive coordinated chromatin motion (Zidovska et al., 2013; Shaban et al., 2018, 2020; Locatelli et al., 2022; Prince et al., 2026). Modeling of nuclear mechanics and shape reveals that RNA Pol II pushing and pulling on DNA during transcriptional activity can generate chromatin motion capable of deforming the nucleus (Liu et al., 2021; Berg et al., 2023). Previous findings suggested that transcriptional activity is necessary for nuclear bleb formation and stabilization (Berg et al., 2023; Prince et al., 2026). Our data provide the novel insight that nuclear blebs are associated with transcription initiation increased relative to elongation (Fig. 4), which leads to the hypothesis that transcription initiation may aid nuclear bleb formation or stabilization. This additional support factor could be local transcription factors associated with transcription initiation including TFIIA, TFIIB, TFIID, TFIIE, TFIIF, and TFIIH (Wade and Struhl, 2008). Our data further confirm that transcription initiation enrichment over elongation in the nuclear bleb is not a result of nuclear rupture as prostate cancer cell lines LNCaP and DU145 show low levels of nuclear rupture (Chu et al., 2025). Further work will be required to understand the role of transcription initiation relative to elongation as being consistently associated with the nuclear bleb.

### Conclusion

Overall, we found that decreased DNA density remains the gold standard for identifying nuclear blebs. While histone proteins decrease in the nuclear bleb, H3 levels showed greater variability than DNA density. Our data refute that this decreased DNA density is due to the histone modification state of H3K9 and H3K27 as levels of euchromatin and heterochromatin are variable across cell lines. Thus, while nuclear blebbing can be modulated through global changes in the histone modification states (Shimamoto et al., 2017; Stephens et al., 2017; 2018; Hobson et al., 2020; Manning et al., 2025), data are lacking for a role of histone modification states in aiding local formation and/or stabilization of the nuclear bleb. On the other hand, we report that transcription initiation is consistently enriched relative to elongation in the nuclear bleb-to-body ratio in a small yet statistically significant manner. This suggests that local enrichment of transcription initiation compared to elongation is associated with stable nuclear blebs. Further research will be needed to determine the role of enriched transcription initiation activity as this finding is currently correlative and lacks mechanistic understanding. Taken together, the histone modification state cannot explain the decreased DNA density that hallmarks nuclear blebs. We found that consistent enrichment of transcription initiation relative to elongation in the nuclear bleb suggests that it is a universal component of nuclear blebs.

## MATERIALS AND METHODS

### Cell culture

MEFs were previously described in the scientific literature (Shimi et al., 2008; Stephens et al., 2018; Vahabikashi et al., 2022). The cells were cultured in DMEM (Corning) supplemented with 10% FBS (HyClone) and 1% penicillin/streptomycin (P/S; Corning), then incubated at 37°C in 5% CO_2_. The cells were passaged after reaching 80-90% confluency or every 2 to 3 days. For passaging, the cells were treated with 0.25% trypsin (Gibco) and 0.1% EDTA without sodium bicarbonate (Corning). Then, the cells were replated and diluted with DMEM. Human fibrosarcoma HT1080 cells, obtained from the American Type Culture Collection (ATCC), were cultured and passaged similarly.

Three human prostate cancer cell lines obtained from the ATCC were used: LNCaP, DU145 and PC3. DU145 and LNCaP cells were cultured in HyClone RPMI 1640 (Cytiva) supplemented with 10% FBS and 1% P/S. PC3 cells were cultured in DMEM supplemented with 10% FBS and 1% P/S.

### Drug treatments

MEFs and HT1080 cells were treated with either 4 mM VPA (1069-66-5, Sigma-Aldrich; Gurvich et al., 2004) or 1 μM DZNep (Cal Biochem; Miranda et al., 2009) for 16 h before time-lapse imaging or fixation for immunofluorescence imaging. PC3 cells were treated with 4 mM VPA for 16 h before fixation for immunofluorescence imaging.

### Time-lapse imaging and analysis

As previously outlined by Manning et al. (2025), images were captured using Nikon Elements software on a Nikon Instruments Ti2-E microscope equipped with a 40× Plan Apo Lambda objective (NA 0.75, WD 0.66, MRH00401) and a 1.5× magnification optivar. Instruments used include the Orca Fusion Gen III camera, Lumencor Aura III light engine, and TMC CleanBench air table. Live-cell time-lapse imaging was conducted using the Nikon Perfect Focus System and Okolab heat, humidity, and CO_2_ stage top incubator (H301). HT1080 NLS-GFP H2B-mCherry cells were imaged in 2-min intervals over ten fields of view for 6 h. Images were captured at 2% power 10-ms exposure time with 480 nm excitation light and 40% power 20-ms exposure time with 560 nm excitation light. Each field of view was observed to measure nuclear body ratios of NLS-GFP and H2B in both rupturing and stable non-rupturing blebbed nuclei using the NIS-Elements AR Analysis software.

### Immunofluorescence

The cells were grown on coverslips in preparation. The cells were fixed with a solution of 4% paraformaldehyde and 0.1% glutaraldehyde in PBS (Corning) for 15 min. Between steps, the cells were washed three times with PBS containing 0.1% Tween 20 and 0.2 g/l azide (PBS-Tw-Az). The cells were permeabilized with 0.5% Triton X-100 in PBS for 10 min, followed by washing with PBS-Tw-Az. Humidity chambers were prepared using Petri dishes, filter paper, sterile distilled water and parafilm. 50 μl of primary antibody solution was applied to the parafilm, and the cell side of the coverslip was placed on top to incubate for 1 h at 37°C in a humidity chamber. The primary antibodies used were H3K27ac mouse MA5-23516 (1:1000, Thermo Fisher Scientific), H3K9me2,3 mouse (1:1000, 5327S, Cell Signaling Technology), H3K27me3 rabbit (1:1000, 9733S, Cell Signaling Technology), and H3K9ac rabbit (1:1000, 9649S, Cell Signaling Technology). For transcription initiation and elongation, we used the previously verified antibodies anti-RNA Pol II CTD repeat YSPTSPS (phospho S5/pSer5) at 1:1000 (Abcam, 4H8 – ChIP Grade, ab5408, mouse monoclonal antibody) and anti-RNA Pol II CTD repeat YSPTSPS (phospho S2/pSer2) at a dilution of 1:1000 (Abcam, ab5095, rabbit polyclonal antibody) (Berg et al., 2023; Prince et al., 2026; Borowski et al., 2026 preprint). The humidity chambers were removed from the incubator, and the coverslips were washed in PBS-Tw-Az. New humidity chambers were made for secondary antibody incubation. 50 μl of secondary antibody was placed on the parafilm, and then the coverslips were placed cell side down and incubated in humidity chambers at 37°C for 30 min. The secondary antibody solution contained goat anti-rabbit 488 (1:200, Cat. No. 4412S, Cell Signaling Technology) and goat anti-mouse 555 (1:200, Cat. No. 4409S, Cell Signaling Technology). Afterwards, coverslips were washed with PBS-Tw-Az and placed cell side down on a glass slide with a drop of mounting medium containing DAPI. Alternatively, cells were stained with a dilution of 1 μg/ml (1:10,000) of Hoechst 33342 (Life Technologies) in PBS for 5 min. Then, the cells were washed with PBS three times and mounted with ProLong Gold Antifade (Invitrogen). The slides were allowed to cure for a day at 4°C before imaging.

### Fluorescence imaging and analysis

As outlined in previous studies (Chiu et al., 2023; Bunner et al., 2025; Chu et al., 2025), immunofluorescence images were acquired using a 12-bit monochrome CCD camera (QICAM Fast 1394, 4.65×4.65 µm pixel size, 1.4 MP, 1392×1040 pixels) with Micromanager and a 40× objective lens on a Nikon TE2000 inverted widefield fluorescence microscope with a CoolLED pE-300 light source. Cells were imaged using transmitted light to find the optimal focus on the field of view to observe nuclear blebs. Ultraviolet light (excitation at 360 nm) was used to visualize DNA via DAPI. Blue and green fluorescence lights (excitation at 480 and 560 nm, respectively) were used to visualize secondary antibodies. 30 fields of view were captured per slide under all three channels. The images were saved and transferred to NIS-Elements (Nikon) or Fiji software for analysis (Schindelin et al., 2012).

Immunofluorescence images of cells stably expressing H2B-mCherry and NLS-GFP as well as immunofluorescence of histone H3 or RNA Pol II pSer5/2 were acquired using a Nikon Instruments Ti2-E microscope with Crest V3 Spinning Disk Confocal, Hamamatsu Orca Fusion Gen III camera, Lumencor Aura III light engine, TMC CleanBench air table, with a 40× air objective (NA 0.75, WD 0.66, MRH00401), and a 12-bit camera through Nikon Elements software. Images were taken at 0.5 μm z-steps over 4.5 μm.

### Nuclear bleb analysis

As outlined previously by Bunner et al. (2025), the images were analyzed in NIS-Elements or exported to Fiji (Schindelin et al., 2012) software to analyze the intensity of each component by normalizing bleb intensity to nuclear body intensity. The nuclear body, bleb, and background of each image were measured by drawing regions of interest (ROIs) via the polygon selection tool. The ROIs of the whole nuclear bleb and whole nuclear body were manually outlined for each nucleus in either the NLS-GFP or DNA (Hoechst 33342) image. The background ROI was a 30×30 pixel square placed in an area with no cells for each image. Measurements of mean intensity for each ROI were recorded and exported to Microsoft Excel. Within Excel, the background intensity was subtracted from the body and bleb. Then the average bleb intensity was divided by the average nuclear body intensity to obtain a relative measurement, where the same average intensity would result in a value of 1. Bleb-to-body ratios were calculated for each marker (NLS-GFP, Hoechst, H3, H3K27ac, H3K9ac, H3K9me2,3, H3K27me3, and RNA Pol II pSer5/2).

### Statistical analysis

Data sets with two dependent measurements in a single nucleus were analyzed for statistical significance using a two-tailed paired Student's *t*-test in Excel. Data sets with three dependent measurements were analyzed for statistical significance using a one-way analysis of variance (ANOVA) with post hoc Tukey using biological triplicate averages. Technical replicates were used for data sets with only one biological replicate. Each figure legend specifies which statistical test was used on each data set. In summary, two-tailed paired Student's *t*-tests were performed on Figs 1 and 4 and Figs S1 and S2. One-way ANOVA tests were performed on Figs 2 and 3.

## Supplementary Material



10.1242/biolopen.062585_sup1 Supplementary information
