# Supplementary material for: Nuclear blebs are composed of variable chromatin states and display increased transcription initiation relative to elongation
Source: Biol Open. 2026 Oct 30;15(11):bio062585. doi: 10.1242/bio.062585 (PMC13750403; doi:10.1242/bio.062585)
Supplement: Supplementary file 1 [file biolopen-15-062585-s1.pdf]

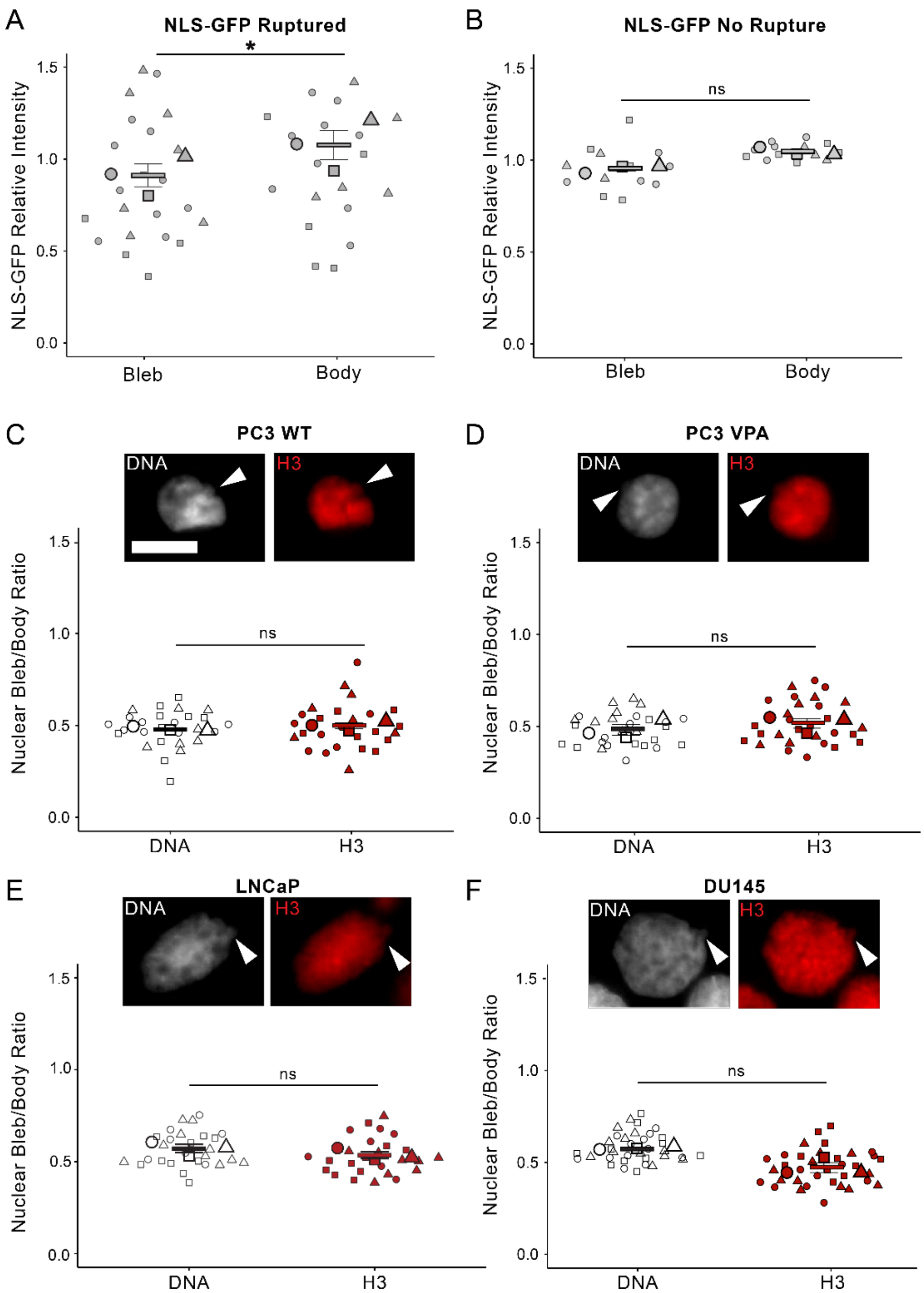

Fig. S1. Histone levels in the nuclear bleb relative to the nuclear body vary across human prostate cancer cell lines. Super plots of NLS-GFP relative intensity in the bleb and body for nuclei that (A) rupture, biological triplicates n = 22, or (B) three nuclei that do not rupture imaged every hour, n = 5, 5, 3. Data analyzed from Figure 1 A and B. (C-F) Example images and super plots of nuclear bleb to body ratio for Hoechst 33342 DNA stain (cyan) and H3 (magenta) for cell lines (C) PC3 n = 9, 10, 8, (D) PC3 VPA n = 10, 9, 10, (E) LNCaP n = 7, 10, 10, (F) DU145 n = 12, 12, 11. All data was collected as biological triplicates. White arrows denote nuclear blebs. Statistical significance is denoted by *P<0.05, **P<0.01, ***P<0.001 or ns (not significant) via two-tailed paired Student's t-test. Scale bar is 10 μm.

Biology Open • Supplementary information

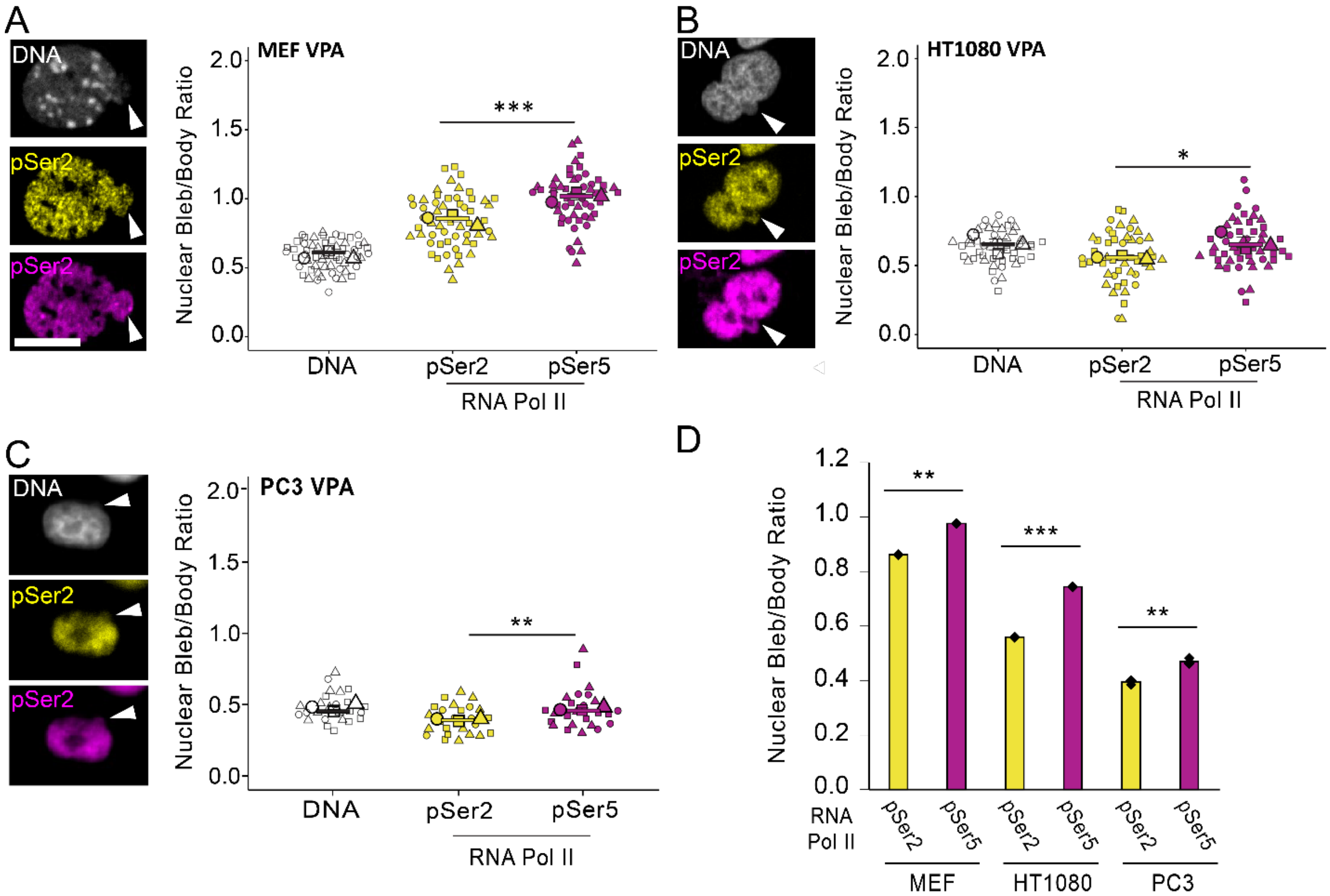

Fig. S2. Active RNA Pol II pSer5 initiation is enriched relative to pSer2 elongation in nuclear blebs of cells treated with VPA. Example images and super plots of nuclear bleb to body ratio for DNA (gray) and RNA Pol II pSer 2 (yellow, elongation) and pSer5 (magenta, initiation) for cell lines treated with histone deacetylase inhibitor VPA 4 mM for 24 hours (A) MEF VPA n = 18, 19, 20; (B) HT1080 VPA n = 10, 20, 20; (C) PC3 VPA n = 6, 9, 10. All are biological triplicates. (D) Graph summarizing all cell lines show a significant enrichment of RNA Pol II pSer5 relative to pSer2 in the nuclear bleb to body ratio. Statistical significance between pSer2 and pSer5 is denoted by **P*<0.05, ***P*<0.01, ****P*<0.001 or ns (not significant) via two-tailed paired Student's *t*-test. Scale bar: 10 µm.

Biology Open • Supplementary information

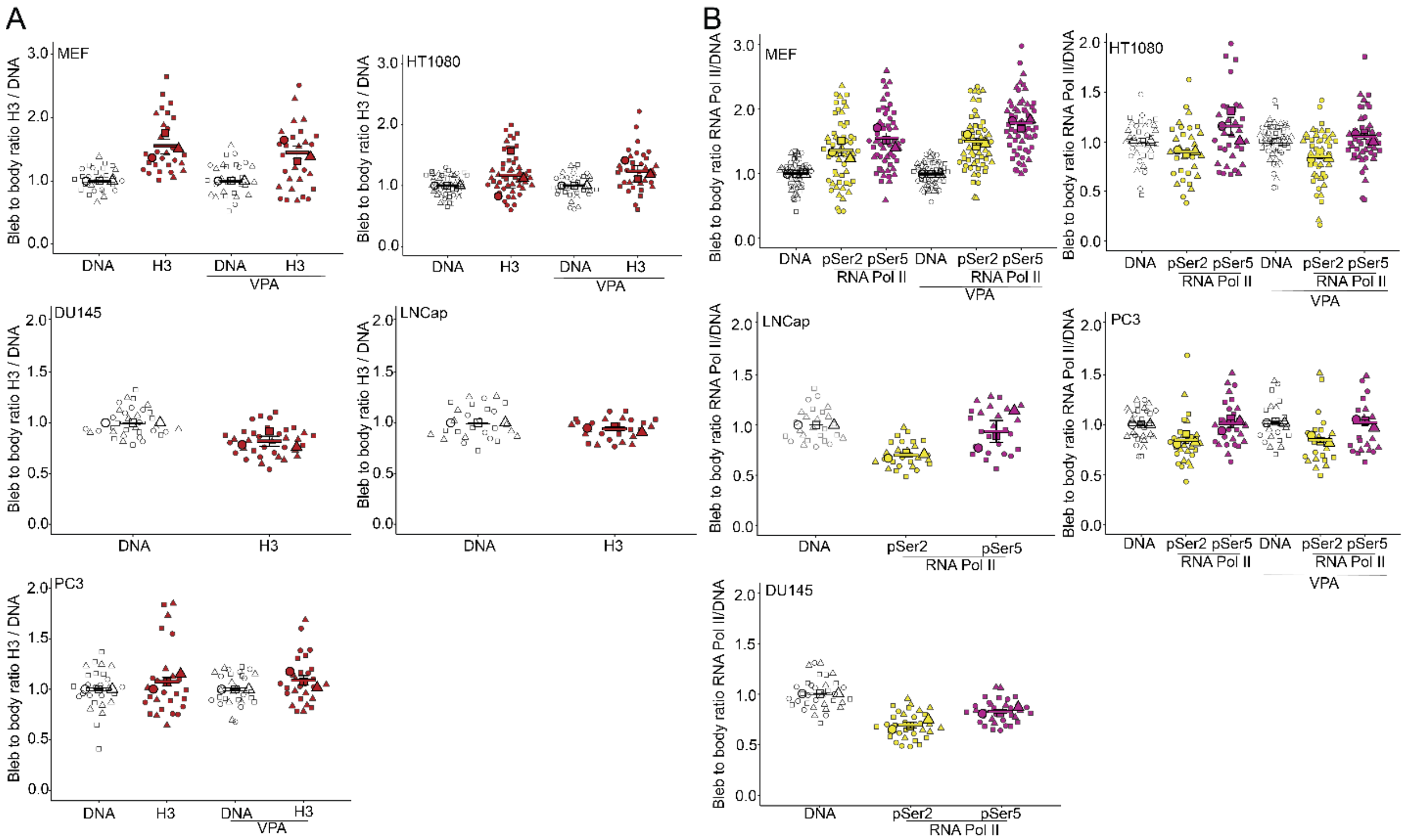

Fig. S3. Summary graphs of bleb to body ratios of H3 and RNA Pol II pSer2/5 relative to DNA. Summary graphs of bleb to body ratios of (A) H3 and (B) RNA Pol II pSer2/5 divided by bleb to body ratios of DNA. Original data for panel A can be found in Fig. 1 and Fig. S1. Original data for panel B can be found in Figure 4. Statistics are the same as original data graphs: (A) figure 1 panels C-D and supplemental 1 panels C-F (B) figure 4 panels A-E.
