# Supplementary material for: Nuclear blebs are composed of variable chromatin states and display increased transcription initiation relative to elongation
Source: Biol Open. 2026 Oct 30;15(11):bio062585. doi: 10.1242/bio.062585 (PMC13750403; doi:10.1242/bio.062585)
Supplement: Supplementary file 2 [file biolopen-15-062585_review_history.pdf]

Nuclear blebs are composed of variable chromatin states and display increased transcription initiation relative to elongation

Madeleine E. Clark, Antonela Losada, Sonia E. Jahng, Allen Saini, Fawwaz A. Chowhan, Gwyneth L. Woods, Adelaide S. Cutler, Stephanie A. Hallerman, Minnah A. Gayed, Sayali R. Bhalerao, Emanuel Bullock, Chris S. Santry, Adriana G. Panagiotou, Bailey Lapolla, Nitish Bhatta, Savanah J. Freidus, Gurnoor Kaur, David Bai, Daniel Hu, Kian Tadbiri, Mary Packard, Katherine Dorfman, Nickolas Borowski, Kelsey Prince, Nick Lang, Carline Fermino do Rosário and Andrew D. Stephens
10.1242/bio.062585

Editor: Christopher A. Maher

Review timeline

Original submission:	23 March 2026
Editorial decision:	3 April 2026
First revision received:	20 August 2026
Editorial decision:	28 August 2026
Second revision received:	3 September 2026
Editorial decision:	21 September 2026
Third revision received:	29 September 2026
Accepted:	30 September 2026

Original submission

First decision letter

MS ID#: bio.062585

MS Title: Nuclear blebs are composed of variable chromatin states but consistently enrich transcription initiation relative to elongation

Authors: Madeleine E. Clark; Antonela Losada; Sonia E. Jahng; Allen Saini; Fawwaz A. Chowhan; Gwyneth L. Woods; Adelaide S. Cutler; Stephanie A. Hallerman; Minnah A. Gayed; Sayali R. Bhalerao; Emanuel Bullock; Chris S. Santry; Adriana G. Panagiotou; Bailey Lapolla; Nitish Bhatta; Savanah J. Freidus; Gurnoor Kaur; David Bai; Daniel Hu; Kian Tadbiri; Mary Packard; Katherine Dorfman; Nickolas Borowski; Kelsey Prince; Nick Lang; Carline Fermino do Rosário; Andrew D. Stephens

I have now reached a decision on the above manuscript.

The reviewer reports are shown at the bottom of this email.
As you will see, the reviewers raised a number of substantial criticisms that prevent me from accepting the paper at this stage.

They suggest, however, that a revised version might prove acceptable, if you can address their concerns. If you think that you can deal satisfactorily with the criticisms on revision, I would be pleased to see a revised manuscript. We would then return it to the reviewers.

At this stage, we also ask you to ensure your manuscript complies with our formatting guidelines. Provided you are able to fully address the referees' comments, we are positive about publication of

© 2026. Published by The Company of Biologists under the terms of the Creative Commons Attribution License (https://creativecommons.org/licenses/by/4.0/).

your paper (we accept over 95% of revision submissions) and therefore hope you won't mind any extra work involved in reformatting your manuscript at this point.

Please upload both a 'clean' version of your Word file, along with a highlighted version clearly showing where you have made changes in the revised manuscript. Please avoid using 'Track changes' in Word files as these are lost in PDF conversion.

I should be grateful if you would also provide a point-by-point response detailing how you have dealt with the points raised by the reviewers in the 'Response to Reviewers' box. Please attend to all of the reviewers' comments. If you do not agree with any of their criticisms or suggestions please explain clearly why this is so.

Reviewer 1

Comments for the author

Summary

The manuscript presented by Clark, Losada et al. reports measurements of some chromatin markers in nuclear blebs in several cell lines. Nuclear blebbing is associated with various cellular disfunctions, and often leads to nuclear rupture and exposure of nucleoplasm and DNA to the cytosolic factors. Here the authors use immunofluorescence and fluorescently tagged proteins to document some possible changes in local nuclear bleb environment relative to the bulk of the nucleoplasm. The authors report that histones H2B and H3 levels are decreased, that euchromatin/heterochromatin are not enriched/depleted and hat RNA Pol II initiation-to-elongation ratio is enriched.

Comments

I think that the manuscript would present a valuable documentation of the DNA status in the nuclear blebs in the measured conditions, if the authors fix some shortcomings (in this reviewer opinion) in presenting results and describing their and others work as commented below.

Overall Approach:

Through the manuscript the authors typically measure ratio of fluorescence of interests (NLS-GFP, DNA stain, fluorescently-labelled antibodies etc) in the bleb and in the nuclear body. And results are usually described as enrichment/depletion of the marker of interest. However, given that the DNA is depleted in the bleb (beginning of the manuscript, and previous work by the Stephens and other labs), it would be more informative if the authors directly report ratio for a marker of interest/DNA for nuclear body and bleb. In such case enrichment/depletion description would make more sense.

On the same note, from the authors' description it is not clear how they defined fluorescence background as it is a crucial aspect for any ratio calculations. Moreover, a better description of how exactly ROI for a bleb and a nuclear body were generated would help to understand better the data. From my personal perspective, I would recommend using multiple ROIs of the same size as a nuclear bleb, to quantify fluorescence in the nuclear body, as many signals from immunofluorescence experiments shown in the paper are very spotty and this will result in a high variation for the signal in the bleb vs signal in the body.

Specific Comments:

For Fig.2, and related section, I have a hard time connecting shown image examples (here, adding outline of what was used as a nuclear bleb and body would be appreciated) with quantification in the plot. For almost all cells shown I cannot see DNA fluorescence in the bleb but rather a strong signal in immunofluorescence… Perhaps, either images are not very representative or quantification have some flows. And most importantly, at the end of the section the authors conclude "the data show that euchromatin is not enriched in the nuclear bleb", while most of the images and plots

© 2026. Published by The Company of Biologists under the terms of the Creative Commons Attribution License (https://creativecommons.org/licenses/by/4.0/).

show trends up and about half of them is significant in the authors' own evaluation. This might be misleading.

Somewhat similar problem is with Fig.3. and related section. Overall we see similar picture - overall trend up with some nominal differences between K9me vs K27me (with only 2 out of 10 conditions passing some significance threshold), with less significant hits, and yet the authors conclude that "facultative heterochromatin shows a bleb to body ratio similar to DNA while constitutive heterochromatin has an increased bleb to body ratio relative to DNA". I don't think this statement correctly reflects data in Fig.3 and 2. Another aspect that the authors didn't explicitly address in their experiments or report is how they deal with heterochromatin being enriched at the nuclear periphery (and one might expect this to hold for nuclear blebs). Importantly, if the authors want to make any claims "relative to DNA", it would be helpful to calculate marker/DNA ration explicitly and compare them (as discussed above).

Same caveat underlines results in Fig.4 and related section, as the authors conclude that "RNA Pol II phosphorylation transcription initiation (pSer5) was consistently increased relative to transcriptional elongation (pSer2) in nuclear blebs". But ratio of pSer5/pSer2 was never examined/plotted, as well as their ratio to the DNA in blebs and the bulk nucleoplasm. We also see that pSer2 and pSer5 signal have very different spatial distribution with pSer2 being much more "granular". I don't know if this is expected, but that can impact fluorescence calculation when they compare fluorescence in a small bleb relative to a big bulk nucleoplasm. The authors also should keep in mind that they are using different antibodies with different non-specific binding and labeled with different fluophores, this may bias observed mean and variance of the fluorescence which may impact statistical score (in a sense it is "comparing oranges to mandarins", close but not the same). By directly comparing their ratio, the authors will mitigate that systematic error. Also, I can not agree with conclusion that "every examined cell line and condition show the same enrichment of transcription initiation relative to elongation in the nuclear bleb, irrespective of its rupture frequency." First, we have no data on rupture frequency to support this general statement, second, most plots in Fig.4 show very similar ratios between pSer2 and pSer5 signals which are also very similar to DNA ratios (and sometime lower values!)

Writing:

While the abstract is well written, the entire manuscript text is in striking (unfortunate) contrast, as it reads more like a lab notebook records or as a very rough first draft (including several "discussion" headings throughout the text, the authors, perhaps, meant them just for themselves), rather than a ready scholarly paper, with many jargons (e.g. "decreased chromatin"), disrupted or missense sentences ("cell lines provide cell lines") and not clear description of what was done ("performed immunofluorescence to measure bleb").

For example:

"We performed immunofluorescence to measure the nuclear bleb relative to the nuclear body",

"To measure both histone and NLS at the same time"

"nuclear blebs are composed of decreased chromatin"

"cancer cell lines provide numerous more cell lines"

Better connection/discussion with works from other labs and from the same lab (as some experiments overlap with previous report from the same group) will also help to appreciate and place results in a bigger picture. For example, when the authors in the introduction state that the composition of blebs is an open question, they cannot avoid mentioning/discussing previous work (a review by Srivtsava at al. Curr.Opin.Cell Biol 2020 might be a good starting point for literature search) as many labs measured various components (DNA, proteins, lipids) in the blebs. Importantly, what is completely ignored through the entire manuscript from intro to conclusions that different etiology of blebs may lead to a different composition.

Minor comments:

© 2026. Published by The Company of Biologists under the terms of the Creative Commons Attribution License (https://creativecommons.org/licenses/by/4.0/).

For fig. S1, I don't quite understand what is plotted in A-B, y-label reads "Relative Intensity" - relative to what?

I would also recommend to show grey scale images for all figures, as the authors want as to compare intensity levels, and this is also a recommended standard for reporting microscopy images.

It is not clear how many biological replicates were measured in what experiments.

Reviewer 2

Comments for the author

This manuscript investigates nuclear blebs in several different cells lines by measuring histone composition and modification states of euchromatin and heterochromatin. This is based on a prior study from the same lab that found nuclear blebs contain decreased DNA density. Using quantitative immunofluorescence and live cell imaging, the authors assess global histone levels, euchromatin and heterochromatin markers within nuclear blebs relative to the nuclear body. The principal conclusions are that (1) decreased DNA density remains the most consistent hallmark of nuclear blebs, (2) local histone modification-defined chromatin states are variable across cell lines and not consistently associated with bleb formation, and (3) transcription initiation is consistently increased in the nuclear bleb relative to elongation.

The study is well done, employs appropriate controls, and addresses an important question with implications for human health. The data support the conclusions, particularly the reproducible enrichment of transcription initiation relative to elongation. Some interpretations, however, would benefit from more cautious framing to avoid overstating causality or generality beyond what is directly measured.

Major Comments

1. The experimental data support the main conclusions. The finding that histone modification-defined chromatin states are variable across blebs is shown across multiple cell lines. However, several discussion statements imply stronger mechanistic conclusions than are shown by the data. The study demonstrates correlation but does not directly establish causality at the level of individual blebs. These conclusions should be framed more cautiously.

2. The manuscript argues that histone modification state is not an essential component of nuclear blebs. While the variability across conditions supports the lack of a strict requirement detectable by immunofluorescence, the data do not exclude chromatin organization below the resolution of immunofluorescence or transient chromatin states during bleb formation that are not captured in fixed samples.

3. The consistent enrichment of RNA Pol II phosphoSer5 relative to phosphoSer2 in nuclear blebs across all cell lines, including those with low rupture frequency, is very interesting. This observation extends prior work. However, I would be careful not to state that transcription initiation drives bleb formation.

4. The experimental methods are generally sound and well described. Minor improvements could help. For example, 1) clarify the number of biological replicates versus total nuclei analyzed; 2) mention validation or prior characterization of the RNA Pol II phospho antibodies under the fixation conditions used; and 3) state clearly that transcriptional activity is inferred from Pol II phosphorylation state, not directly measured via RNA synthesis studies.

5. Some typographical and grammatical errors appear in the Results and figure legends (e.g., missing spaces, inconsistent capitalization). Scale bars (plural) are mentioned in the figure caption, but each figure seems to only have a single scale bar.

© 2026. Published by The Company of Biologists under the terms of the Creative Commons Attribution License (https://creativecommons.org/licenses/by/4.0/).

6. The manuscript addresses a meaningful question with clear experimental results and offers a valuable contribution to the field. Clarifying the limits of causal inference and slightly tempering some conclusions will strengthen the manuscript without requiring any additional experiments.

Reviewer's Responses to Questions

Experimental quality

Does each figure have the proper controls?
If 'No', please indicate reasons in Comments for Author box below.
Reviewer #1:
- Yes

Reviewer #2:
- Yes

Were the data analyzed using appropriate statistical tests?
If 'No', please indicate reasons in Comments for Author box below.
Reviewer #1:
- Yes

Reviewer #2:
- Yes

Reproducibility
Were experiments performed using adequate number of biological replicates?
If 'No', please indicate reasons in Comments for Author box below.
Reviewer #1:
- No

Reviewer #2:
- Yes

Does the methods section provide sufficient detail to permit reproducibility?
If 'No', please indicate reasons in Comments for Author box below.
Reviewer #1:
- No

Reviewer #2:
- Yes

Completeness
Are the manuscript's conclusions supported by the data?
If 'No', please indicate reasons in Comments for Author box below.
Reviewer #1:
- No

Reviewer #2:
- Yes

Scholarship
Do the authors cite and discuss the merits of data that would argue for and against their conclusion?

© 2026. Published by The Company of Biologists under the terms of the Creative Commons Attribution License (https://creativecommons.org/licenses/by/4.0/).

If 'No', please indicate reasons in Comments for Author box below.
Reviewer #1:
- No

Reviewer #2:
- Yes

Does the manuscript title & abstract accurately reflect the contents of the manuscript, without hyperbole?
If 'No', please indicate reasons in Comments for Author box below.
Reviewer #1:
- No

Reviewer #2:
- Yes

First revision

Author response to reviewers' comments

Comments from the Reviewers:

Reviewer 1: Summary
The manuscript presented by Clark, Losada et al. reports measurements of some chromatin markers in nuclear blebs in several cell lines. Nuclear blebbing is associated with various cellular disfunctions, and often leads to nuclear rupture and exposure of nucleoplasm and DNA to the cytosolic factors. Here the authors use immunofluorescence and fluorescently tagged proteins to document some possible changes in local nuclear bleb environment relative to the bulk of the nucleoplasm. The authors report that histones H2B and H3 levels are decreased, that euchromatin/heterochromatin are not enriched/depleted and hat RNA Pol II initiation- to-elongation ratio is enriched.

Comments
I think that the manuscript would present a valuable documentation of the DNA status in the nuclear blebs in the measured conditions, if the authors fix some shortcomings (in this reviewer opinion) in presenting results and describing their and others work as commented below.

We appreciate hearing that the reviewer thinks our manuscript "presents a valuable documentation". Below we address the reviewer points.

Overall Approach:

Through the manuscript the authors typically measure ratio of fluorescence of interests (NLS-GFP, DNA stain, fluorescently-labelled antibodies etc) in the bleb and in the nuclear body. And results are usually described as enrichment/depletion of the marker of interest. However, given that the DNA is depleted in the bleb (beginning of the manuscript, and previous work by the Stephens and other labs), it would be more informative if the authors directly report ratio for a marker of interest/DNA for nuclear body and bleb. In such case enrichment/depletion description would make more sense.

Overall, the reviewer looking for A) clarification concerning statements of an immunofluorescence marker's increase or decrease and B) a suggestion for how to address via a marker/DNA ratio. We provide a response for each point.

A) We believe the confusion may be when we say enriched or depleted, that we need to

© 2026. Published by The Company of Biologists under the terms of the Creative Commons Attribution License (https://creativecommons.org/licenses/by/4.0/).

clarify the comparison. To help clarify enrichment and depletion, we have revised the manuscript to clearly state if we are talking about the nuclear bleb relative to the nuclear body OR marker nuclear bleb/body ratio relative to DNA nuclear bleb/body ratio.

B) Comparing nuclear bleb to body ratios of one stain or Immunofluorescence marker compared to another is an established and published comparison (Chu et al., 2026 JCS and Bunner et al. 2025 JCS). As shown in the original manuscript, having DNA bleb/body ratio compared to marker of interest bleb/body ration provides the ability to do statistics. Since all measurements of DNA and marker of interest come from the same blebbed nucleus, no new insights are gained from making a marker of interest/DNA ratio. To directly address the reviewer's point, we added a summary graph showing the requested bleb to body marker/DNA ratio in Figures 2 and 3 as panel D as well as added this marker/DNA ratio in new Supplemental Figure 3 for the data in Figures 1 and 4. This new ratio of bleb to body marker/DNA shows the same statistics as the original data of separate nuclear bleb to body ratio of marker compared to DNA as shown in the original manuscript main figures.

On the same note, from the authors' description it is not clear how they defined fluorescence background as it is a crucial aspect for any ratio calculations. Moreover, a better description of how exactly ROI for a bleb and a nuclear body were generated would help to understand better the data.

In the original manuscript the Materials and Methods stated. "The nuclear body, bleb, and background of each nucleus image were measured by drawing regions of interest (ROI) via the polygon selection tool." This method was cited from our previous publication, Bunner et al., 2025 JCS.

We have revised the materials and methods to better communicate our established methodology from our many previous publications. We now add: "The ROI of the whole nuclear bleb and whole nuclear body were hand drawn for each nucleus. The ROI for the background was square 30X30 pixels placed in an area with no cells for each image."

These measurements come with a high level of control because every measurement of nuclear bleb to body is done within the same image keeping fluorescence setting and background the same between both nuclear bleb and body for a given nucleus.

From my personal perspective, I would recommend using multiple ROIs of the same size as a nuclear bleb, to quantify fluorescence in the nuclear body, as many signals from immunofluorescence experiments shown in the paper are very spotty and this will result in a high variation for the signal in the bleb vs signal in the body.

1) We have established a clear methodology of selecting the whole nuclear bleb vs. the whole nuclear body as previously published (Stephens et al., 2018 MBoC, Bunner et al., 2025 JCS, Chu et al., 2026 JCS). It is unnecessary to change an established published methodology.

2) Nuclear bleb size varies. Thus, taking a nuclear bleb sized ROI and applying it to the nuclear body would require decisions on best placement of multiple ROIs. Placement of a smaller ROI in a larger nuclear body would only increase the variation that the reviewer is worried about. The variation would also be highly dependent on placement. Adding up a multiple smaller ROIs placed throughout the body is effectively the same as drawing an ROI for the entire body. There is no clearly logical benefit to the suggested strategy, and thus we will stay with our well-published methodology mentioned in point 1 above.

3) DNA fluorescent intensity in MEFs is a highly "spotty" signal due to chromocenter foci. However, decreased DNA density is the single most consistent measured stain/immunofluorescence marker through this and many other publications (Stephens et al., 2018 MBoC, Bunner et al., 2025 JCS, Chu et al., 2026 JCS). Thus, the assumption that spottiness is a problem has no data to support it and instead plenty of data refuting it.

Specific Comments:

© 2026. Published by The Company of Biologists under the terms of the Creative Commons Attribution License (https://creativecommons.org/licenses/by/4.0/).

For Fig.2, and related section, I have a hard time connecting shown image examples (here, adding outline of what was used as a nuclear bleb and body would be appreciated) with quantification in the plot.

The reviewer cites their troubles seeing where the nuclear bleb is in each image. To address this problem we have revised the figure to add a white arrow as we did in Figure 1 to denote the nuclear bleb as well as add text to the figure legend.

For almost all cells shown I cannot see DNA fluorescence in the bleb but rather a strong signal in immunofluorescence… Perhaps, either images are not very representative or quantification have some flows.

The reviewer's comment agrees nicely with the quantified data. The inability of the reviewer to see DNA in the nuclear bleb is because DNA is decreased in the bleb relative to the nuclear body while many but not all immunofluorescence markers are not depleted in the nuclear bleb relative to the body.

And most importantly, at the end of the section the authors conclude "the data show that euchromatin is not enriched in the nuclear bleb", while most of the images and plots show trends up and about half of them is significant in the authors' own evaluation. This might be misleading.

The reviewer points out that our wording is confusing. We thank the reviewer for this feedback and have revised the manuscript as we outline below. This confusion is driven by some key missing words. We have revised the text to clarify that "Overall, the data show that euchromatin is not enriched in the nuclear bleb relative to the nuclear body, as most nuclear bleb to body average ratios are not greater than 1". We then continue: "Furthermore, euchromatin is not even consistently higher than decreased DNA density in the nuclear bleb relative to the body." Which refers the fact that MEF WT, HT1080 WT, HT1080 DZNep and DU145 show at least one euchromatin marker that is not statistically different than DNA nuclear bleb to body ratio.

The next paragraph emphasizes the importance of data relative to our hypothesis. "The hypothesis that decreased DNA density in the nuclear bleb is due to euchromatin enrichment is not supported by our data."

Somewhat similar problem is with Fig.3. and related section. Overall we see similar picture - overall trend up with some nominal differences between K9me vs K27me (with only 2 out of 10 conditions passing some significance threshold), with less significant hits, and yet the authors conclude that "facultative heterochromatin shows a bleb to body ratio similar to DNA while constitutive heterochromatin has an increased bleb to body ratio relative to DNA". I don't think this statement correctly reflects data in Fig.3 and 2.

We agree with the reviewer about an overall up trend in the Figure 3 graphs from left to right ordered DNA, H3K27me3, and H3K9me2,3. This is important because our hypothesis was that depletion of heterochromatin in the nuclear bleb would be similar as DNA. As stated in the first line of this results section, "Nuclear blebs' decreased chromatin density could arise from loss of heterochromatin." Thus, the data that H3K9me2,3 nuclear bleb to body ratio increases in 9 of 10 conditions relative to DNA bleb to body ratio completely refutes the hypothesis that decreased DNA density is due to heterochromatin depletion.

The data supports our statement. Let's start with the data shown in Figure 3 and the statement the reviewer pulled from our original manuscript. **"facultative heterochromatin shows a bleb to body ratio similar to DNA…"** H3K27me3 is <u>like</u> DNA nuclear bleb to body ratio for 9 of the 10 conditions. **"…while constitutive heterochromatin has an increased bleb to body ratio relative to DNA".** H3K9me2,3 is <u>statistically significantly increased</u> from DNA nuclear bleb to body ratio for 9 of the 10 conditions. In short, the data do support the statement.

Another aspect that the authors didn't explicitly address in their experiments or report is how they deal with heterochromatin being enriched at the nuclear periphery (and one might expect this to hold for nuclear blebs). Importantly, if the authors want to make any claims

© 2026. Published by The Company of Biologists under the terms of the Creative Commons Attribution License (https://creativecommons.org/licenses/by/4.0/).

"relative to DNA", it would be helpful to calculate marker/DNA ration explicitly and compare them (as discussed above).

The reviewer questions how we dealt with heterochromatin enrichment at the periphery. In short, measurement of peripheral heterochromatin is included in our bulk measurements of nuclear bleb and body, but we do not measure it specifically because that was not the focus of our manuscript. Further studies would be needed to meaningfully address this question that is beyond the current scope of the manuscript.

1) Facultative heterochromatin does not enrich at the periphery; thus measurement of the periphery would be a niche question only for H3K9me3.
2) Enrichment of heterochromatin at the periphery is cell type dependent. In MEFs constitutive heterochromatin is also enriched at the periphery and at chromocenters that are randomly scattered in the nuclear interior. <u>To best measure all sources and locations of heterochromatin across all cell types we used bulk analysis of the whole nuclear bleb and whole nuclear body</u>.
3) Proper analysis of heterochromatin nuclear periphery measurements requires different approaches than our current data acquisition and analysis. Proper measurement of specifically peripheral enriched heterochromatin requires use of spinning disk confocal microscopy. To ask the specific question would require a completely different experimental approach beyond the scope of this manuscript. We again reiterate that the periphery was measured in our bulk nuclear bleb and body ratios.

Below we provide a few example images of H3K9me3 confocal images. H3K9me3 peripheral staining in the nuclear bleb vs. body is highly variable with about half showing maintenance (left column) and half showing loss (right column) in the nuclear bleb. This initial data underscores this question is beyond the current scope.

© 2026. Published by The Company of Biologists under the terms of the Creative Commons Attribution License (https://creativecommons.org/licenses/by/4.0/).

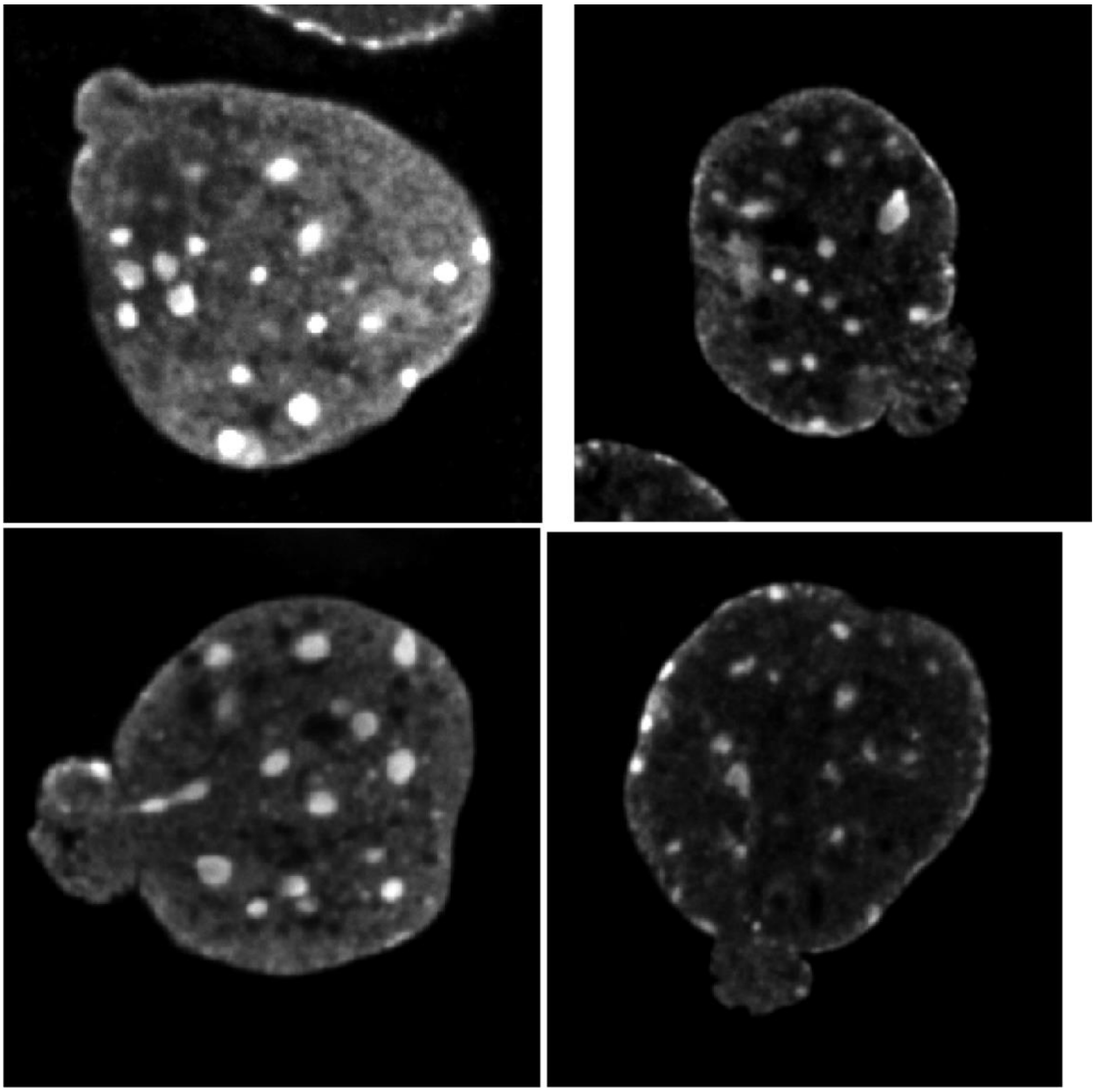

The reviewer questions how we make **“any claims "relative to DNA"**. In the original manuscript we compare DNA nuclear bleb to body ratios with each immunofluorescence marker to quantitatively and statistically determine “relative to DNA” whether it changes or not. We would like to clarify all the data is paired, DNA, H3K27me3, and H3K9me2,3 data is taken from the same nucleus and compared in a paired fashion. Thus, this requested marker/DNA ratio for the nuclear bleb to body ratio is regraphing the same data in a slightly different manner. We now provide the requested bleb to body maker/DNA ratio in panel D of Figures 2 and 3 as well as Supplemental Figure 3 which graphs the original data from Figures 1 and 4 as this requested marker/DNA ratio. This requested ratio gives the same statistics as our nuclear bleb to body ratios in the main figures. The methodology to compare DNA nuclear bleb to body ratio relative to another markers bleb to body ratio has been previously published twice in the sister journal JCS (Bunner et al 2025 and Cu et al 2026).

Same caveat underlines results in Fig.4 and related section, as the authors conclude that "RNA Pol II phosphorylation transcription initiation (pSer5) was consistently increased relative to transcriptional elongation (pSer2) in nuclear blebs". But ratio of pSer5/pSer2 was never examined/plotted, as well as their ratio to the DNA in blebs and the bulk nucleoplasm.

First the reviewer’s statement of **“But ratio of pSer5/pSer2 was never examined/plotted.”** In the original manuscript, we plotted data for nuclear bleb to body ratio for pSer5 vs. pSer2 in Figure 4 across 5 cell lines as well as upon VPA treatment shown in Supplemental Figure 2. Examination of these measurements showed a statistically significant difference for all cell lines measured. Thus, we did plot and examine.

© 2026. Published by The Company of Biologists under the terms of the Creative Commons Attribution License (https://creativecommons.org/licenses/by/4.0/).

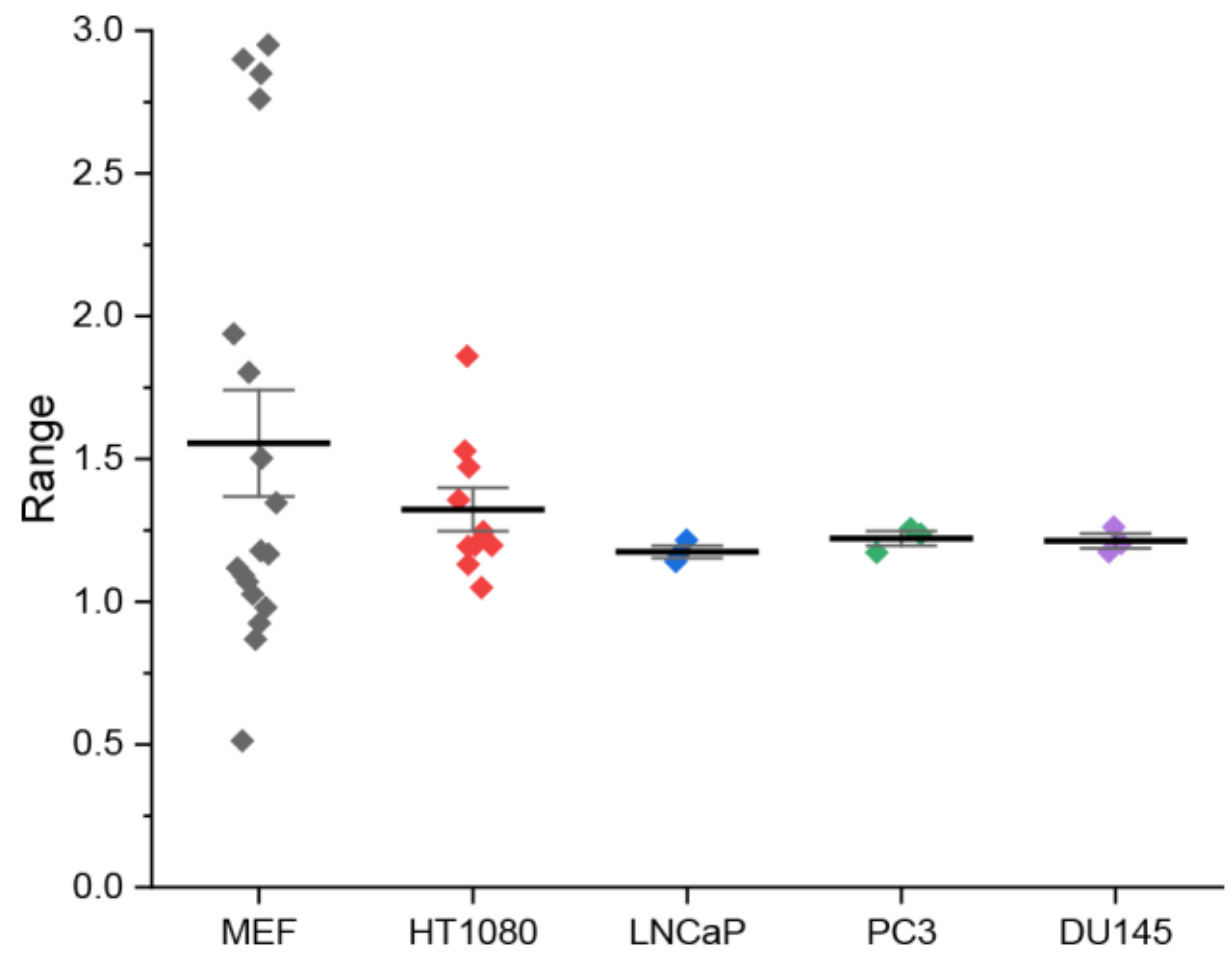

We now include nuclear bleb to body ratio of pSer5 and pSer2 divided by DNA in Supplemental Figure 3. Again, this is regraphing that provides nothing new for the reader as it leads to the same statistical outcomes. Similarly, making a visual representation of Ser5p/Ser2p (see below) would only provide qualitative data in which statistics and conclusions could not be drawn. It is possible that using the standard error bars not touching 1 shows significance. Here we include a graph of Ser5/2p that confirms our original findings that Ser5p is higher than Ser2p of RAN Pol II. It is worth noting that all imaged blebbed nuclei were stained with DNA and RNA Pol II pSer5 and pSer2, providing paired data.

We also see that pSer2 and pSer5 signal have very different spatial distribution with pSer2 being much more "granular". I don't know if this is expected, but that can impact fluorescence calculation when they compare fluorescence in a small bleb relative to a big bulk nucleoplasm.

The reviewer is concerned about the possible effect of different signals. All of our immunofluorescence markers have some level of differences. Decreased DNA density is the most consistent and reproducible, but DNA signal has foci due to chromocenters in MEF nuclei (making them more" granular") but no chromocenter foci occur in the human cell lines. While these two cell lines produce different visible patterns, both produce highly reproducible bleb to body data. This data refutes concerns over perceived granular images. Out of all the immunofluorescence markers including Histone H2B, H3, H3K27ac, H3K9ac, H3K27me3, H3K9me2,3, RNA Pol II pSer5/2 – the only one that shows a consistent trend is RNA Pol II pSer5 vs. pSer2.

The authors also should keep in mind that they are using different antibodies with different non-specific binding and labeled with different fluophores, this may bias observed mean and variance of the fluorescence which may impact statistical score (in a sense it is "comparing oranges to mandarins", close but not the same). By directly comparing their ratio, the authors will mitigate that systematic error.

The reviewer is worried about A) different antibodies having different non-specific binding and B) using the requested marker/DNA ratio.

A) Our data directly accounts for variability of antibody behavior. Measurement of nuclear bleb vs. nuclear body fluorescence are both background subtracted the same because they are in the same image. Thus, our fluorescence measurements account for variability. If an antibody binds less well in the bleb, it will also bind less well in the body, and it binds nonspecifically more or less that will be accounted for by background subtract applied the same to both the bleb and body. Thus, our approach addresses and removes this error.

B) We have generated the requested bleb to body marker/DNA ratio which results in the same statistical analysis as the original data. This is expected as they come from the same cored data.

Also, I can not agree with conclusion that "every examined cell line and condition show the

© 2026. Published by The Company of Biologists under the terms of the Creative Commons Attribution License (https://creativecommons.org/licenses/by/4.0/).

same enrichment of transcription initiation relative to elongation in the nuclear bleb, irrespective of its rupture frequency." First, we have no data on rupture frequency to support this general statement, second, most plots in Fig.4 show very similar ratios between pSer2 and pSer5 signals which are also very similar to DNA ratios (and sometime lower values!)

Overall, the reviewer raises concerns over 1) rupture frequency data and 2) comparison of pSer5 vs. 2.

1. In the revised manuscript we clarify that rupture frequency data comes from previously published papers (Chu, Lang et al., 2025 JCB). We have revised the manuscript to clarify this point by adding: "LNCap and Du145 cells rarely rupture whereas PC3 cells rupture frequently similar to MEF and HT1080 cells (Chu et al., 2025)."
2. Statistics of two-tailed paired Students t-test reveals that all measured cell lines show a significant increase in RNA Pol II pSer5 relative to pSer2. Both immunofluorescence markers were imaged in the same nuclear bleb and body. While we measured many immunofluorescence markers, RNA Pol II pSer5 increase relative to pSer2 was the only measurement that was consistent across multiple cell lines and conditions (Figure 4 and Supplemental Figure 2). Of course, besides the already established decreased DNA density in the nuclear bleb.
3. The hypothesis that histone modification-based chromatin compaction underlies nuclear blebbing was refuted because not all conditions showed increased euchromatin and decreased heterochromatin relative to DNA. An alternative hypothesis for consistency of nuclear bleb composition would be the other major contributor, transcriptional activity. While this exact finding of differential RNA Pol II activity levels (via pSer5/2) does not have a mechanism, the finding is consistent whereas other markers remain variable.

Writing:
While the abstract is well written, the entire manuscript text is in striking (unfortunate) contrast, as it reads more like a lab notebook records or as a very rough first draft (including several "discussion" headings throughout the text, the authors, perhaps, meant them just for themselves), rather than a ready scholarly paper, with many jargons (e.g. "decreased chromatin"), disrupted or missense sentences ("cell lines provide cell lines") and not clear description of what was done ("performed immunofluorescence to measure bleb").

We have revised the text to make this manuscript publication worthy. Open Biology has an open submission format in which we felt that clearly denoting the discussion sections was helpful for reviewers. We appreciate the feedback and thus, we have removed the discussion subtitles, as requested by the reviewer. Furthermore, we know have a proper discussion section in the manuscript. Below we address each point.

"decreased chromatin" Has been revised to say "less compact chromatin, DNA and the proteins that compact it." Therefore, we provide a definition of chromatin the first time it is used. We have revised this throughout the manuscript.

"cell lines provide cell lines" We have revised this sentence to now say: "We used three human prostate cancer cell lines that provide different nuclear bleb rupture incidence rates." We would like to reiterate the differential rupture frequency was established in a recent publication (Chu, Lang et al., 2025 JCS). We cite this publication in the following sentence.

"performed immunofluorescence to measure bleb" We have revised the manuscript to state: "To determine the composition of nuclear blebs within the context of histones and histone modification states, we performed immunofluorescence and measured the fluorescent signal in the nuclear bleb relative to the nuclear body across multiple cell lines."

For example:
"We performed immunofluorescence to measure the nuclear bleb relative to the nuclear body", We have revised the text to state " We then conducted immunofluorescence and fluorescence imaging experiments to compare bleb to body ratios of DNA to histone H3."

© 2026. Published by The Company of Biologists under the terms of the Creative Commons Attribution License (https://creativecommons.org/licenses/by/4.0/).

"To measure both histone and NLS at the same time" We have revised the text to state " To simultaneously measure both, we imaged histone H2B-mCherry and NLS-GFP in stably expressing HT1080 cells lines."
"nuclear blebs are composed of decreased chromatin" We have revised the text to state " Overall, this data supports the hypothesis that nuclear blebs are composed of less compact chromatin, DNA and the proteins that compact it."

"cancer cell lines provide numerous more cell lines" We have revised the text to state "We used three human prostate cancer cell lines that provide different nuclear bleb rupture incidence rates. Blebbed nuclei in PC3 cells rupture frequently, similar to MEF and HT1080 cells, whereas LNCaP and DU145 rarely rupture (Chu et al., 2025)."

Better connection/discussion with works from other labs and from the same lab (as some experiments overlap with previous report from the same group) will also help to appreciate and place results in a bigger picture. For example, when the authors in the introduction state that the composition of blebs is an open question, they cannot avoid mentioning/discussing previous work (a review by Srivtsava at al. Curr.Opin.Cell Biol 2020 might be a good starting point for literature search) as many labs measured various components (DNA, proteins, lipids) in the blebs. Importantly, what is completely ignored through the entire manuscript from intro to conclusions that different etiology of blebs may lead to a different composition.

To address the reviewers point we have added the citation to **Srivtsava at al. Curr.Opin.Cell Biol 2021** as a source of general information as requested by the reviewer. This review is a fantastic example of the importance of our manuscript. The section discussing heterochromatin and euchromatin in this review only discuss theoretical changes but provides no citations of primary data measuring levels in the nuclear bleb.
***Alternatively, we cite two publications with actual previous measurements of eu/heterochromatin in nuclear blebs. We appreciate the feedback to add this review because it highlights the novelty of our work relative to the lack of data that exists measuring eu/heterochromatin levels in nuclear blebs.

The introduction is meant to teach the reader about the content essential to the manuscript's story. Our manuscript focuses on chromatin through histone proteins (H2B and H3), histone modification state, and transcription activity as denoted specifically by RNA Pol II phosphorylation. Thus, we believe a review of all of the studies of nuclear bleb composition is unnecessary.

The reviewer states that we have ignored etiology, or the origin of the nuclear blebs which could cause different nuclear bleb compositions. We appreciate the feedback. We have added a discussion paragraph to the revised manuscript directly addressing this point of etiology. We provide some of the points below:

Overall, it is our intent to ignore the origin or causes of the bleb to determine the universal or essential components of a nuclear bleb. Proof that we can find universal components exist in DNA density (Bunner et al 2025), loss of lamin B1 due to nuclear rupture (Chu et al 2026), and our novel data for differences in Ser5p vs. Ser2p in RNA Pol II (Figure 4).

While we appreciate the reviewer's opinion, there is no data to support that etiology matters. Nuclear blebs that form in wild type, increased euchromatin (VPA), decreased heterochromatin (DZNEP), loss of lamin B1 (Lmnb1 -/-) and lamin A KD (LA KD) all require both actin confinement and contraction (Stephens et al 2018; Pho et al 2024, as well as numerous others) and/or perturbations decrease nuclear mechanics (Stephens et al 2018 and others), and rely on active transcription (Berg et al. 2023, Prince et al. 2025).

Minor comments:
For fig. S1, I don't quite understand what is plotted in A-B, y-label reads "Relative Intensity" - relative to what?

We have revised Supplemental Figure 1 y axis to state "NLS-GFP relative intensity".

© 2026. Published by The Company of Biologists under the terms of the Creative Commons Attribution License (https://creativecommons.org/licenses/by/4.0/).

I would also recommend to show grey scale images for all figures, as the authors want as to compare intensity levels, and this is also a recommended standard for reporting microscopy images.

As this is a minor comment, we believe this is stylistic decision.

It is not clear how many biological replicates were measured in what experiments. We have revised the text to more clearly report number of biological replicates in each figure legend.

Reviewer 2: This manuscript investigates nuclear blebs in several different cells lines by measuring histone composition and modification states of euchromatin and heterochromatin. This is based on a prior study from the same lab that found nuclear blebs contain decreased DNA density. Using quantitative immunofluorescence and live cell imaging, the authors assess global histone levels, euchromatin and heterochromatin markers within nuclear blebs relative to the nuclear body. The principal conclusions are that (1) decreased DNA density remains the most consistent hallmark of nuclear blebs, (2) local histone modification-defined chromatin states are variable across cell lines and not consistently associated with bleb formation, and (3) transcription initiation is consistently increased in the nuclear bleb relative to elongation. The study is well done, employs appropriate controls, and addresses an important question with implications for human health. The data support the conclusions, particularly the reproducible enrichment of transcription initiation relative to elongation. Some interpretations, however, would benefit from more cautious framing to avoid overstating causality or generality beyond what is directly measured.

Major Comments

1. The experimental data support the main conclusions. The finding that histone modification-defined chromatin states are variable across blebs is shown across multiple cell lines. However, several discussion statements imply stronger mechanistic conclusions than are shown by the data. The study demonstrates correlation but does not directly establish causality at the level of individual blebs. These conclusions should be framed more cautiously.

We thank the reviewer for clearly stating that the data support our main conclusions. We have revised the text to more carefully discuss these findings in each discussion section.

2. The manuscript argues that histone modification state is not an essential component of nuclear blebs. While the variability across conditions supports the lack of a strict requirement detectable by immunofluorescence, the data do not exclude chromatin organization below the resolution of immunofluorescence or transient chromatin states during bleb formation that are not captured in fixed samples.

We thank the reviewer for providing interesting discussion points that we have incorporated into the revised manuscript. We add that "Our data does not account for histone modification state during nuclear formation which might rely on more mobile euchromatin."

3. The consistent enrichment of RNA Pol II phosphoSer5 relative to phosphoSer2 in nuclear blebs across all cell lines, including those with low rupture frequency, is very interesting. This observation extends prior work. However, I would be careful not to state that transcription initiation drives bleb formation.

We have revised the text to remove statements alluding to transcription initiation drives bleb formation. We agree with the reviewer, as our past data states that cells with transcription activity suppressed can still form nuclear blebs but those blebs are short- lived.

4. The experimental methods are generally sound and well described. Minor improvements could help. For example, 1) clarify the number of biological replicates versus total nuclei analyzed; 2) mention validation or prior characterization of the RNA Pol II phospho antibodies

© 2026. Published by The Company of Biologists under the terms of the Creative Commons Attribution License (https://creativecommons.org/licenses/by/4.0/).

under the fixation conditions used; and 3) state clearly that transcriptional activity is inferred from Pol II phosphorylation state, not directly measured via RNA synthesis studies.

We have addressed each of the points raised by the reviewer.

1. Bio replicates – In the revised manuscript we have now added number of biological replicates for each figure legend along with number of measurements per replicate.
2. RNA Pol II pSer5/2 IF conditions – In the revised manuscript we have now added a sentence addressing the immunofluorescence of active RNA Pol II. "In the revised manuscript we have now added "For transcription initiation and elongation, the previously verified antibodies anti-RNA pol II CTD repeat YSPTSPS (phospho S5/pSer5) at 1:1000 (Abcam, 4H8 – ChIP Grade, ab5408, mouse monoclonal antibody) and anti-RNA pol II CTD repeat YSPTSPS (phospho S2/pSer2) at 1:1000 (Abcam, ab5095, rabbit polyclonal antibody)(Berg et al., 2023; Prince et al., 2025; Borowski et al., 2026)."
3. Inferred RNA transcription activity - In the revised manuscript we clarify that "Transcriptional activity can be inferred through immunofluorescence measurements of active RNA Pol II phosphorylated at Serine 5 for initiation and Serine 2 for elongation…"

5. Some typographical and grammatical errors appear in the Results and figure legends (e.g., missing spaces, inconsistent capitalization). Scale bars (plural) are mentioned in the figure caption, but each figure seems to only have a single scale bar.

We have revised the manuscript to fix spelling errors, inconsistent capitalization, and mention of scale bar.

6. The manuscript addresses a meaningful question with clear experimental results and offers a valuable contribution to the field. Clarifying the limits of causal inference and slightly tempering some conclusions will strengthen the manuscript without requiring any additional experiments.

We thank the reviewer for their helpful feedback and have made the suggested revisions to strengthen the manuscript.

<u>Second decision letter</u>

MS ID#: bio.062585R1

MS Title: Nuclear blebs are composed of variable chromatin states but consistently enrich transcription initiation relative to elongation

Authors: Madeleine E. Clark; Antonela Losada; Sonia E. Jahng; Allen Saini; Fawwaz A. Chowhan; Gwyneth L. Woods; Adelaide S. Cutler; Stephanie A. Hallerman; Minnah A. Gayed; Sayali R. Bhalerao; Emanuel Bullock; Chris S. Santry; Adriana G. Panagiotou; Bailey Lapolla; Nitish Bhatta; Savanah J. Freidus; Gurnoor Kaur; David Bai; Daniel Hu; Kian Tadbiri; Mary Packard; Katherine Dorfman; Nickolas Borowski; Kelsey Prince; Nick Lang; Carline Fermino do Rosário; Andrew D. Stephens

I have now reached a decision on the above manuscript.

The reviewer reports are shown at the bottom of this email.

As you will see, the reviewers gave favourable reports, but raised some critical points that will require amendments to your manuscript. I hope that you will be able to carry these out, because we would like to be able to accept your paper.

© 2026. Published by The Company of Biologists under the terms of the Creative Commons Attribution License (https://creativecommons.org/licenses/by/4.0/).

At this stage, we also ask you to ensure your manuscript complies with our formatting guidelines – please see our manuscript preparation guidelines for details. Provided you are able to fully address the referees' comments, we are positive about publication of your paper (we accept over 95% of revision submissions) and therefore hope you won't mind any extra work involved in reformatting your manuscript at this point.

Please upload both a 'clean' version of your Word file, along with a highlighted version clearly showing where you have made changes in the revised manuscript. Please avoid using 'Track changes' in Word files as these are lost in PDF conversion.

I should be grateful if you would also provide a point-by-point response detailing how you have dealt with the points raised by the reviewers in the 'Response to Reviewers' box. Please attend to all of the reviewers' comments. If you do not agree with any of their criticisms or suggestions please explain clearly why this is so.

Reviewer 1

Comments for the author

The revised manuscript is greatly improved in readability and added previously missing details concerned with image analysis. As in original manuscripts the authors reports measurements of some chromatin markers in nuclear blebs in several cell lines; and concluded that, in blebs, histones H2B and H3 levels are decreased, that euchromatin/heterochromatin levels are not consistently enriched/depleted and that RNA Pol II initiation-to-elongation ratio is enriched.

General Comments

While the manuscript greatly improved, I still believe that there are some concerns to be addressed to avoid confusion/misinterpretation of the results.

1. I think that the title is misleading and doesn't reflect results presented. Firstly, active "nuclear blebs enrich" (especially in contrast with passive tense "are composed") implies that nuclear blebs by some mechanism actively changing initiation/elongation ratio but there is no evidence for that (i.e. we have no knowledge of the dynamic of this ratio after bleb appearance). Secondly, there are some reasonable doubts about whether this ratio is significantly changed (see below).

2. The main claim of the paper is that the elongation is enriched relative to the elongation. However, this "yes/no" type of statement doesn't fully reflect the authors observation. Firstly, if it was true, I think it is important to note that the effect is modest at best (for example, ~15% for MEF cells, and this is likely to be an overestimate, see note on image analysis). Secondly, Fig.4F is not consistent with 4A - in F we see that mean pSer5 level is ~1.0, while it in A it is ~0.9. Thirdly, and most importantly, if we take mean values of 3 biological replicates (acknowledgements to the authors for providing data) for pSer2 and pSer5 for MEF cells, as the biological replicates are independent measurements, but cells from the same biological replicate are not - we get, using t-test, that null hypothesis (that pSer2 and pSer5 have the same mean value) cannot be rejected ($p \sim 0.22$).

3. And again, if the authors make claim about initiation/elongation ratio change in bleb vs nuclear body, it would be better to directly calculate this ratio of pSer2/pSer5 signals in the bleb and compare it to the one in the body, not to compare ratio bleb/body ratio of different signals.

One of the reasons is that the measured signal S is the sum of background, non-specific fluorescence (non-specific binding, autofluorescence, etc) and specific signal (of interest, but it also has different amplifications for different antibodies, i.e. monoclonal vs polyclonal, or different fluophores): $S = S_b + S_{ns} + S_{sp}$. While the authors subtracted S_b, they don't have any estimates of non-specific binding and amplification, i.e. of S_{ns}. And this can quite dramatically influence ratio calculations, the ratio $(S_{ns} + S_{sp})_{body}$/ $(S_{ns} + S_{sp})_{nuc}$ will strongly depend on relative values of S_{ns} vs S_{sp} that may have a big change for different antibodies and fluophores.

© 2026. Published by The Company of Biologists under the terms of the Creative Commons Attribution License (https://creativecommons.org/licenses/by/4.0/).

Unfortunately, the authors didn't provide measurements of intensities for different ROI, only the ratio results.

4. Thank you for providing details on image analysis. But there are a couple important details that are still missing. First, while ROI outlines were drawn manually, based on what is still not specified (DNA signal? Brightfield? Some protein marker signal?). Second, as it was drawn manually, did authors tried to test whether there is a human bias in the results - i.e. did they try blind test and more than one person for the same datasets? It wouldn't be important if changes were big, but given small observed differences it becomes important.

Reviewer 2

Comments for the author

The authors have addressed most of my concerns satisfactorily, particularly the methodological clarifications and many, but not all, of the requests for more cautious interpretation. However, there remain a few areas where the revised manuscript still overstates conclusions relative to the data, and these should be addressed before acceptance.

The manuscript is scientifically sound, and the revisions have substantially improved transparency and clarity. Most of my concerns were addressed adequately. Nevertheless, several statements in the discussion still state stronger conclusions than is warranted by the data.

There are still overstated mechanistic conclusions and references to causality. The data support the conclusions, but the manuscript should not imply mechanistic causality because the observations are correlative. 1) For example, "Our data provides novel insight that specifically transcription initiation may support either nuclear bleb formation or stabilization." and "Peripheral and highly crosslinked constitutive heterochromatin could be a driving force for nuclear blebs." My recommendation is that the Discussion should frame these as hypotheses rather than interpretations supported by the current data. 2) Immunofluorescence cannot exclude unresolved chromatin organization or transient states. The data do not exclude chromatin organization below the resolution of immunofluorescence or transient chromatin states during bleb formation. 3) The should avoid claiming that transcription initiation drives bleb formation, as they've done here "specifically transcription initiation may support either nuclear bleb formation or stabilization" because that still suggests a mechanistic role. The data only support that pSer5 is enriched relative to pSer2 in blebs. They do not support that pSer5 contributes to formation or stabilization. I suggest that the authors replace mechanistic wording with "is associated with" throughout the discussion.

I think that the etiology discussion remains problematic. The authors added a paragraph regarding etiology. However, the statement: "there is no data to support that etiology matters" is not scientifically justified. The current dataset does not test whether etiology affects composition. I recommend deleting or substantially revising this section.

They note that "Technical replicates were used for data sets with only one biological replicate." Figure 4 includes conditions where only one biological replicate was collected. This is a limitation that should be acknowledged when discussing reproducibility of the RNA Pol II findings. It is not necessarily grounds for additional experimentation, but it is worth noting.

If these revisions are made, I would support publication.

Reviewer's Responses to Questions

Experimental quality
Are experiments performed using appropriate methods that will answer the question (or test the hypothesis or support the observations) posed by the authors? Is the right tool used for the job?
If 'No', please indicate reasons in Comments for Author box below.
Reviewer #1:

- Yes

© 2026. Published by The Company of Biologists under the terms of the Creative Commons Attribution License (https://creativecommons.org/licenses/by/4.0/).

Reviewer #2:
- Yes

Were the data analyzed using appropriate statistical tests?
If 'No', please indicate reasons in Comments for Author box below.
Reviewer #1:
- Yes

Reviewer #2:
- Yes

Reproducibility
Were experiments performed using adequate number of biological replicates?
If 'No', please indicate reasons in Comments for Author box below.
Reviewer #1:
- Yes

Reviewer #2:
- No

Is there sufficient raw data to assess the rigor of the analysis?
If 'No', please indicate reasons in Comments for Author box below.
Reviewer #1:
- No

Reviewer #2:
- Yes

Does the methods section provide sufficient detail to permit reproducibility?
If 'No', please indicate reasons in Comments for Author box below.
Reviewer #1:
- No

Reviewer #2:
- Yes

Completeness
Are the manuscript's conclusions supported by the data?
If 'No', please indicate reasons in Comments for Author box below.
Reviewer #1:
- No

Reviewer #2:
- Yes

Scholarship
Do the authors cite and discuss the merits of data that would argue for and against their conclusion?
If 'No', please indicate reasons in Comments for Author box below.
Reviewer #1:
- Yes

© 2026. Published by The Company of Biologists under the terms of the Creative Commons Attribution License (https://creativecommons.org/licenses/by/4.0/).

Reviewer #2:
- Yes

Does the manuscript title & abstract accurately reflect the contents of the manuscript, without hyperbole?
If 'No', please indicate reasons in Comments for Author box below.
Reviewer #1:
- No

Reviewer #2:
- Yes

Second revision

Author response to reviewers' comments

Comments from the Reviewers:

Reviewer 1:

Summary

The revised manuscript is greatly improved in readability and added previously missing details concerned with image analysis. As in original manuscripts the authors reports measurements of some chromatin markers in nuclear blebs in several cell lines; and concluded that, in blebs, histones H2B and H3 levels are decreased, that euchromatin/heterochromatin levels are not consistently enriched/depleted and that RNA Pol II initiation-to-elongation ratio is enriched.

General Comments

While the manuscript greatly improved, I still believe that there are some concerns to be addressed to avoid confusion/misinterpretation of the results.

1. I think that the title is misleading and doesn't reflect results presented. Firstly, active "nuclear blebs enrich" (especially in contrast with passive tense "are composed") implies that nuclear blebs by some mechanism actively changing initiation/elongation ratio but there is no evidence for that (i.e. we have no knowledge of the dynamic of this ratio after bleb appearance). Secondly, there are some reasonable doubts about whether this ratio is significantly changed (see below).

We have revised the title to "Nuclear blebs are composed of variable chromatin states and increased transcription initiation relative to elongation".

Additional measurements in the revision verify that the ratio in question is indeed statistically significant for increased pSer5 relative to pSer2 in the nuclear bleb (see below).

2. The main claim of the paper is that the elongation is enriched relative to the elongation. However, this "yes/no" type of statement doesn't fully reflect the authors observation. Firstly, if it was true, I think it is important to note that the effect is modest at best (for example, ~15% for MEF cells, and this is likely to be an overestimate, see note on image analysis). Secondly, Fig.4F is not consistent with 4A - in F we see that mean pSer5 level is ~1.0, while it in A it is ~0.9. Thirdly, and most importantly, if we take mean values of 3 biological replicates (acknowledgements to the authors for providing data) for pSer2 and pSer5 for MEF cells, as the biological replicates are independent measurements, but cells from the same biological replicate are not - we get, using t-test, that null hypothesis (that pSer2 and pSer5 have the same mean value) cannot be rejected (p~0.22).

© 2026. Published by The Company of Biologists under the terms of the Creative Commons Attribution License (https://creativecommons.org/licenses/by/4.0/).

Overall the reviewer raises the question of statistical significance for RNA Pol II pSer5 vs pSer2, which we address by adding new data that reports statistical significance.

1. We agree that the change is small but statistically significant. We have added text to the revised manuscript to reflect this point: "All measured changes in the nuclear bleb to body ratio of RNA Pol II pSer5 relative to pSer2 are both small in percentage and statistically significant **(Fig. 4F)**."
2. We have fixed Figure 4F to reflect the newly added data to Figure 4A-B.
3. Figure 4A data came from 2 biological replicates, the second of which was split into two technical replicates. First, in the new Figure 4 A we kept all the data for the second biological replicate together. To test the statistical significance of the MEF biological replicates, we performed two more biological replicates to gather new data. Thus, we now provided data for 4 biological replicates to address the reviewer's concern. This data confirms that RNA Pol II pSer5vs. pSer2 bleb to body ratio is statistically significantly different for both a two-tailed paired students t-test ($p = 0.041$) and a One Way Anova (0.0006). We would like to note that a two-tailed parried students t-test run on every single biological replicate individually ($n = 18$-40) showed statistically significant differences for RNA Pol II pSer5vs. pSer2 bleb to body ratio, as previously noted by the reviewer.

Overall, we appreciate the reviewer's attention to detail which has pushed us to have proper levels of biological replicates that report statistically significant differences between RNA Pol II pSer5vs. pSer2 bleb to body ratio. The point #3 requested ratio also shows statistical significance.

3. And again, if the authors make claim about initiation/elongation ratio change in bleb vs nuclear body, it would be better to directly calculate this ratio of pSer2/pSer5 signals in the bleb and compare it to the one in the body, not to compare ratio bleb/body ratio of different signals.

One of the reasons is that the measured signal S is the sum of background, non- specific fluorescence (non-specific binding, autofluorescence, etc) and specific signal (of interest, but it also has different amplifications for different antibodies, i.e. monoclonal vs polyclonal, or different fluophores): S = S_b + S_ns + S_sp. While the authors subtracted S_b, they don't have any estimates of non-specific binding and amplification, i.e. of S_ns. And this can quite dramatically influence ratio calculations, the ratio (S_ns + S_sp)_body/ (S_ns + S_sp)_nuc will strongly depend on relative values of S_ns vs S_sp that may have a big change for different antibodies and fluophores. Unfortunately, the authors didn't provide measurements of intensities for different ROI, only the ratio results.

We provide the requested ratio of RNA Pol II pSer5/2 in the bleb vs. body for MEFs. This calculation, which is derived from the exact same data used to make our established nuclear bleb/body ratio, also provides statistical significance (see below).

© 2026. Published by The Company of Biologists under the terms of the Creative Commons Attribution License (https://creativecommons.org/licenses/by/4.0/).

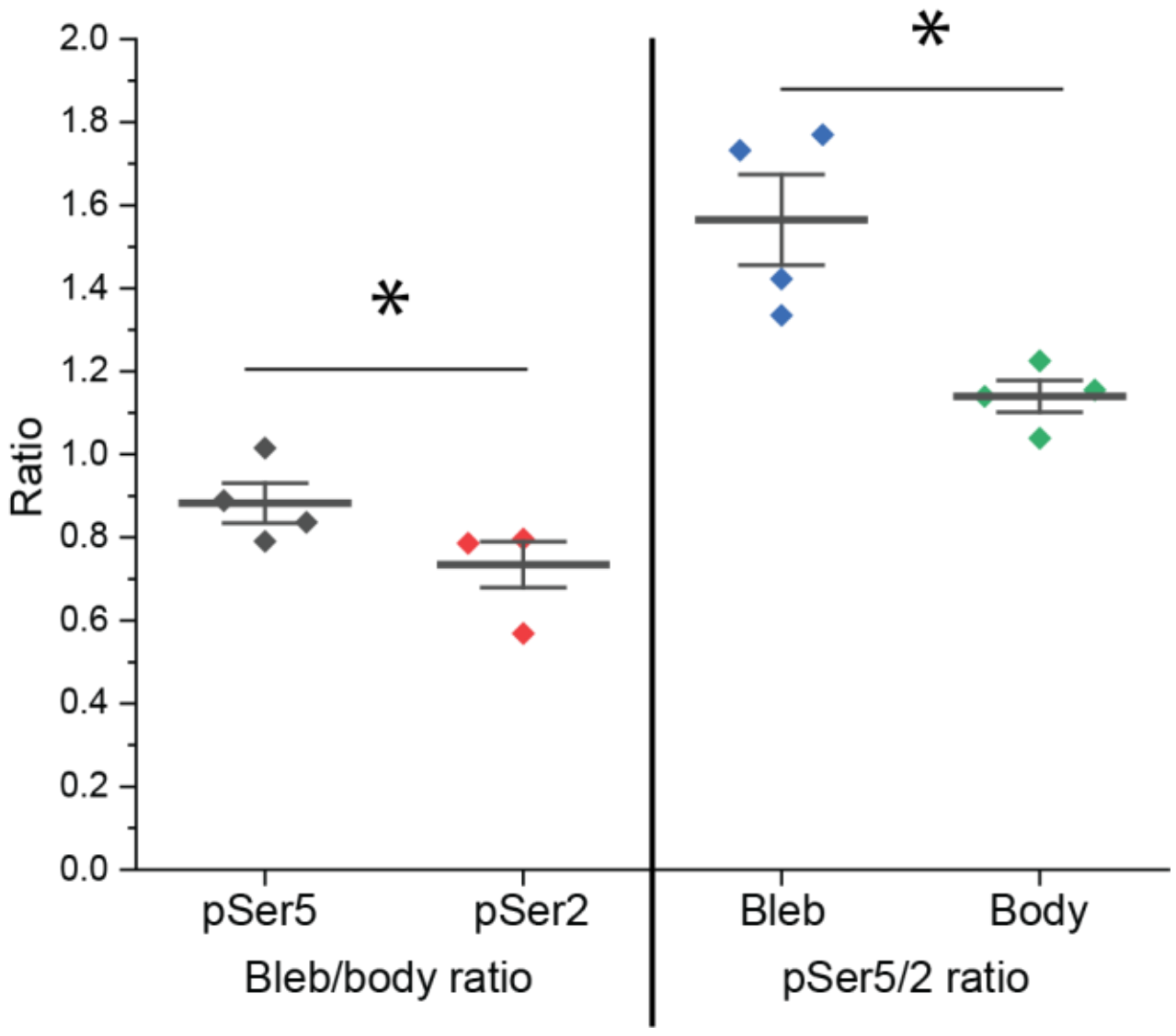

The reviewer raises the same question we answered previously about different antibodies, fluorophores, and non-specific binding. Clearly put the reviewer's **S = S_b + S_ns + S_sp** is the same for the nuclear bleb and body thus normalizing and removing the exact concerns of the reviewer. As we stated in the first round of reviews, for the past two publications (Bunner et al., AND Chu et al., JCS) our established nuclear bleb to body ratio normalizes the data for any given antibodies, fluorophores, and non-specific binding. It does this by using the same image to calculate differences between bleb and body in the same channel (fluorophore). Any non-specific binding or fluorophore "problem" would be the same in the bleb and the body and thus accounted for by our approach.

4. Thank you for providing details on image analysis. But there are a couple important details that are still missing. First, while ROI outlines were drawn manually, based on what is still not specified (DNA signal? Brightfield? Some protein marker signal?). Second, as it was drawn manually, did authors tried to test whether there is a human bias in the results - i.e. did they try blind test and more than one person for the same datasets? It wouldn't be important if changes were big, but given small observed differences it becomes important.

We have revised the manuscript to state: " The ROI of the whole nuclear bleb and whole nuclear body were hand drawn for each nucleus in either the NLS-GFP or DNA (Hoechst 33342) image."

Human bias is a systematic error that applies similarly to wild type vs. other conditions as well as nuclear bleb and body. The proof that systematic error does not drastically affect the measurements is that decreased DNA is consistently found in all cell lines and conditions independent of the human drawing the ROI over this paper and all of our previous papers.

The statement that small differences would matter for human bias is refuted by the data. The data for pSer5/2 was measured by 3 different groups of 2-3 people across multiple cell lines and conditions and showed consistent statistical significance, in contrast to euchromatin and heterochromatin measurements. Again, the data support that human systematic error, even for small differences, is insignificant.

Reviewer 2: The authors have addressed most of my concerns satisfactorily, particularly the methodological clarifications and many, but not all, of the requests for more cautious interpretation. However, there remain a few areas where the revised manuscript still overstates conclusions relative to the data, and these should be addressed before acceptance.

The manuscript is scientifically sound, and the revisions have substantially improved transparency and clarity. Most of my concerns were addressed adequately.

© 2026. Published by The Company of Biologists under the terms of the Creative Commons Attribution License (https://creativecommons.org/licenses/by/4.0/).

Nevertheless, several statements in the discussion still state stronger conclusions than is warranted by the data.

We would like to point out that the reviewer stated: “**The manuscript is scientifically sound**”. We have revised the manuscript’s discussion to tone down statements flagged by the reviewer.

There are still overstated mechanistic conclusions and references to causality. The data support the conclusions, but the manuscript should not imply mechanistic causality because the observations are correlative.

1) For example, "Our data provides novel insight that specifically transcription initiation may support either nuclear bleb formation or stabilization."and "Peripheral and highly crosslinked constitutive heterochromatin could be a driving force for nuclear blebs." My recommendation is that the Discussion should frame these as hypotheses rather than interpretations supported by the current data.

We have revised the manuscript to clearly note that these are hypotheses.

“Our data provides the novel insight that nuclear blebs are associated with transcription initiation increased relative to elongation (**Fig. 4**) which leads to the hypothesis that transcription initiation may aid nuclear bleb formation or stabilization.”

“An alternative hypothesis to be tested in the future is that peripheral and highly crosslinked constitutive heterochromatin could be a driving force for nuclear blebs. This idea is supported by our data’s agreement with physics simulations showing that peripheral heterochromatin enrichment might be required for nuclear blebbing (Attar *et al.*, 2024).”

2) Immunofluorescence cannot exclude unresolved chromatin organization or transient states. The data do not exclude chromatin organization below the resolution of immunofluorescence or transient chromatin states during bleb formation.

We have revised the manuscript to include this idea. “Our immunofluorescence data does not account for histone modification state below the widefield resolution limit or transient chromatin states during nuclear bleb formation.”

3) The should avoid claiming that transcription initiation drives bleb formation, as they've done here "specifically transcription initiation may support either nuclear bleb formation or stabilization" because that still suggests a mechanistic role. The data only support that pSer5 is enriched relative to pSer2 in blebs. They do not support that pSer5 contributes to formation or stabilization. I suggest that the authors replace mechanistic wording with "is associated with" throughout the discussion.

We have revised this sentence in the manuscript: “Our data provides the novel insight that nuclear blebs are associated with transcription initiation increased relative to elongation (**Fig. 4**) which leads to the hypothesis that transcription initiation may aid nuclear bleb formation or stabilization.”

I think that the etiology discussion remains problematic. The authors added a paragraph regarding etiology. However, the statement: "there is no data to support that etiology matters" is not scientifically justified. The current dataset does not test whether etiology affects composition. I recommend deleting or substantially revising this section.

We have removed this statement and the revised statement is now: “However, an ever- mounting list of evidence suggests that all nuclear blebs share key characteristics.” The reviewer is correct that we do not have data ruling out etiology. Instead this paragraph focuses on shared characteristics seen consistently in all nuclear blebs independent of cell type or condition.

They note that "Technical replicates were used for data sets with only one biological replicate." Figure 4 includes conditions where only one biological replicate was collected. This is a limitation that should be acknowledged when discussing reproducibility of the RNA Pol II

© 2026. Published by The Company of Biologists under the terms of the Creative Commons Attribution License (https://creativecommons.org/licenses/by/4.0/).

findings. It is not necessarily grounds for additional experimentation, but it is worth noting.
The reviewer points out a mistake in the text. We have fixed the Figure 4 legend to accurately state that we do have biological triplicates for all measured cell lines.

"for cell lines (A) MEF n = 18, 40, 20,18; (B) HT1080 n = 10, 10, 14; (C) LNCaP n = 6, 10, 10, (D), PC3 n = 9, 7, 13, (E) DU145 n = 12, 11, 11. All are biological triplicates or more."

If these revisions are made, I would support publication.

Third decision letter

MS ID#: bio.062585R2

MS Title: Nuclear blebs are composed of variable chromatin states and increased transcription initiation relative to elongation

Authors: Madeleine E. Clark; Antonela Losada; Sonia E. Jahng; Allen Saini; Fawwaz A. Chowhan; Gwyneth L. Woods; Adelaide S. Cutler; Stephanie A. Hallerman; Minnah A. Gayed; Sayali R. Bhalerao; Emanuel Bullock; Chris S. Santry; Adriana G. Panagiotou; Bailey Lapolla; Nitish Bhatta; Savanah J. Freidus; Gurnoor Kaur; David Bai; Daniel Hu; Kian Tadbiri; Mary Packard; Katherine Dorfman; Nickolas Borowski; Kelsey Prince; Nick Lang; Carline Fermino do Rosário; Andrew D. Stephens

I have now reached a decision on the above manuscript.

The reviewer report is shown at the bottom of this email.

As you will see, the reviewer gave a favourable report, but raised some critical points that will require amendments to your manuscript. I hope that you will be able to carry these out, because we would like to be able to accept your paper.

At this stage, we also ask you to ensure your manuscript complies with our formatting guidelines - please see our manuscript preparation guidelines for details. Provided you are able to fully address the referees' comments, we are positive about publication of your paper (we accept over 95% of revision submissions) and therefore hope you won't mind any extra work involved in reformatting your manuscript at this point.

Please upload both a 'clean' version of your Word file, along with a highlighted version clearly showing where you have made changes in the revised manuscript. Please avoid using 'Track changes' in Word files as these are lost in PDF conversion.

I should be grateful if you would also provide a point-by-point response detailing how you have dealt with the points raised by the reviewers in the 'Response to Reviewers' box. Please attend to all of the reviewers' comments. If you do not agree with any of their criticisms or suggestions please explain clearly why this is so.

Reviewer 1

In summary, There are still few concerns that, in this reviewer's opinion, should be addressed before the publication. Overall, all the concerns are about the authors' wording and can be easily addressed. If these revisions are made (to the editor's discretion, no need for additional review), I would support publication.

Title "Nuclear blebs are composed of variable chromatin states and increased transcription initiation relative to elongation" While the authors tried to address the concern about the title, the

© 2026. Published by The Company of Biologists under the terms of the Creative Commons Attribution License (https://creativecommons.org/licenses/by/4.0/).

title is still confusing. It has two potential readings: “Nuclear blebs are <> increased transcription initiation relative to elongation”, which is very confusing. Or, “Nuclear blebs <> increased transcription initiation relative to elongation”, Which would be overstatement not supported by the reported results.

pSer5/2 ratio results I think that “we report that transcription initiation is consistently enriched relative to elongation in the nuclear bleb to body ratio”, while supported by presented data (though see a note below) does not accurately represent results. The authors partially addressed this by including “All measured changes in the nuclear bleb to body ratio of RNA Pol II pSer5 relative to pSer2 are both small in percentage and statistically significant”. But it was followed by much stronger last conclusive statement: “Taken together, active RNA Pol II transcription initiation was consistently increased compared to transcription elongation in nuclear blebs across all measured cell lines”.

I strongly believe that notion that effect is qualitatively small has to be added in summarizing statements – in abstract, end of the pSer2/5 section in Results and in Conclusion. Otherwise, if the readers don’t look carefully in the figures, may not realize magnitude of the effect.

Despite the author’s response, there are still concerns whether statistically significant for reported values is equivalent to meaningful in this context (that is why caution in the statements is advised). First, if measured signal can described as $S_{pser} = S_b + S_{ns} + S_{sp}$, then normalizing and taking ration does not take care of S_{ns}. Say $S_b = 0$ (by substracting background outside of the cell) and let say $S_{ns} = 1$ and 5 for pSer2 and pSer5, respectively, while signals are the same, $S_{sp} =$ 5 and 5. Then, $S_{pSer2}/S_{pSer5} = (1+5)/(5+5) = 0.6$ instead of real ratio equal 1. Second, the authors own plot in the response for pSer2/pSer5 ratio shows very different effect: in the left panel, the ratio is ~1.25, while in the right panel ~1.4, while naively we expect that it should be the same even with unresolved issue of the contribution from non-specific binding/fluorescence (if this expectation is incorrect, please, explain in the manuscript).

Facultative vs Constitutive Heterochromatin results

In this section every, but the first, paragraph has conclusive statements that are not fully supported by the authors’ own statistical analysis: p.2: “Thus, relative to DNA MEF cells reveal that facultative and constitutive heterochromatin have different levels in nuclear blebs relative to their nuclear body.” Fig.3A actually states that the difference between K27me and K9me is insignificant (meaning inconclusive). p.3: “Taken together, constitutive heterochromatin is not depleted in nuclear bleb relative to the nuclear body while facultative heterochromatin is depleted”, implying that there is a difference between K27me and K9me ratios. But Fig.3B says that the differences are not significant. p.4: “Over multiple cell lines facultative heterochromatin shows similarly depleted bleb to body ratios as DNA, while constitutive heterochromatin has an increased bleb to body ratio relative to DNA.”, again interpreting results as K27me and K9me ratios are different overall, while 8/11 shown panels showed insignificant differences.

Reviewer's Responses to Questions

Experimental quality
Are experiments performed using appropriate methods that will answer the question (or test the hypothesis or support the observations) posed by the authors? Is the right tool used for the job?
If 'No', please indicate reasons in Comments for Author box below.
Reviewer #1:

- Yes

Were the data analyzed using appropriate statistical tests?
If 'No', please indicate reasons in Comments for Author box below.
Reviewer #1:

- Yes

© 2026. Published by The Company of Biologists under the terms of the Creative Commons Attribution License (https://creativecommons.org/licenses/by/4.0/).

Reproducibility
Were experiments performed using adequate number of biological replicates?
If 'No', please indicate reasons in Comments for Author box below.
Reviewer #1:
- Yes

Is there sufficient raw data to assess the rigor of the analysis?
If 'No', please indicate reasons in Comments for Author box below.
Reviewer #1:
- Yes

Does the methods section provide sufficient detail to permit reproducibility?
If 'No', please indicate reasons in Comments for Author box below.
Reviewer #1:
- Yes

Completeness
Are the manuscript's conclusions supported by the data?
If 'No', please indicate reasons in Comments for Author box below.
Reviewer #1:
- No

Scholarship
Do the authors cite and discuss the merits of data that would argue for and against their conclusion?
If 'No', please indicate reasons in Comments for Author box below.
Reviewer #1:
- Yes

Does the manuscript title & abstract accurately reflect the contents of the manuscript, without hyperbole?
If 'No', please indicate reasons in Comments for Author box below.
Reviewer #1:
- No

Third revision

Author response to reviewers' comments

The authors addressed one of the important concerns about one of their main result by increasing statistics for measurements of pSer5/2 ratio in MEF cells. There are still few concerns that, in this reviewer's opinion, should be addressed before the publication.

Response: The reviewer points out that we accomplished a major requested revision thought supporting the statistics for measurements of pSer5/2.

Overall, all the concerns are about the authors' wording and can be easily addressed. If these revisions are made (to the editors discretion, no need for additional review), I would support publication.

© 2026. Published by The Company of Biologists under the terms of the Creative Commons Attribution License (https://creativecommons.org/licenses/by/4.0/).

Response: We have addressed the wording issues below point-by-point and in the revised manuscript.

Title

"Nuclear blebs are composed of variable chromatin states and increased transcription initiation relative to elongation". While the authors tried to address the concern about the title, the title is still confusing. It has two potential readings: "Nuclear blebs are <> increased transcription initiation relative to elongation", which is very confusing. Or, "Nuclear blebs <> increased transcription initiation relative to elongation", Which would be overstatement not supported by the reported results.

Response: We have added the word "display" to address the reviewer's concern. The Title in the revised manuscript is now: "Nuclear blebs are composed of variable chromatin states and display increased transcription initiation relative to elongation"

pSer5/2 ratio results

I think that "we report that transcription initiation is consistently enriched relative to elongation in the nuclear bleb to body ratio", while supported by presented data (though see a note below) does not accurately represent results. The authors partially addressed this by including "All measured changes in the nuclear bleb to body ratio of RNA Pol II pSer5 relative to pSer2 are both small in percentage and statistically significant". But it was followed by much stronger last conclusive statement: "Taken together, active RNA Pol II transcription initiation was consistently increased compared to transcription elongation in nuclear blebs across all measured cell lines".

I strongly believe that notion that effect is qualitatively small has to be added in summarizing statements - in abstract, end of the pSer2/5 section in Results and in Conclusion. Otherwise, if the readers don't look carefully in the figures, may not realize magnitude of the effect.

Response: We have added that it is small but statistically significant to the summary sentence as well. The revised sentence at the end of the section in the results as follows: "Taken together, active RNA Pol II transcription initiation was consistently increased compared to transcription elongation in nuclear blebs across all measured cell lines in a small yet statistically significant manner." Also, we add this to the abstract and conclusion, as requested.

Despite the author's response, there are still concerns whether statistically significant for reported values is equivalent to meaningful in this context (that is why caution in the statements is advised). First, if measured signal can be described as *S_pser* = *S_b* + *S_ns* + *S_sp*, then normalizing and taking ration does not take care of *S_ns*. Say *S_b* = 0 (by substracting background outside of the cell) and let say *S_ns* = 1 and 5 for pSer2 and pSer5, respectively, while signals are the same, *S_sp* = 5 and 5. Then, *S_pSer2/S_ pSer5* = (1+5)/(5+5) = 0.6 instead of real ratio equal 1. Second, the authors own plot in the response for pSer2/pSer5 ratio shows very different effect: in the left panel, the ratio is ~1.25, while in the right panel ~1.4, while naively we expect that it should be the same even with unresolved issue of the contribution from non- specific binding/fluorescence (if this expectation is incorrect, please, explain in the manuscript).

Response: In science statistical significance is how data are reported to either change or not. The reviewer is asking for an equation that considers non-specific binding. The data in the manuscript is calculated as bleb to body ratio which takes non-specific binding into account because it is the same across the cell, or more specifically the nuclear bleb and body. The referred to plot in the response ask for a different approach was requested by a reviewer and is not the basis for conclusions in the manuscript. Again, the bleb to body measurement is an established approach from multiple publications (Bunner et al., AND Chu et al., JCS).

Facultative vs Constitutive Heterochromatin results

© 2026. Published by The Company of Biologists under the terms of the Creative Commons Attribution License (https://creativecommons.org/licenses/by/4.0/).

In this section every, but the first, paragraph has conclusive statements that are not fully supported by the authors' own statistical analysis:

p.2: "Thus, relative to DNA MEF cells reveal that facultative and constitutive heterochromatin have different levels in nuclear blebs relative to their nuclear body."

Fig.3A actually states that the difference between K27me and K9me is insignificant (meaning inconclusive).

p.3: "Taken together, constitutive heterochromatin is not depleted in nuclear bleb relative to the nuclear body while facultative heterochromatin is depleted", implying that there is a difference between K27me and K9me ratios. But Fig.3B says that the differences are not significant.

p.4: "Over multiple cell lines facultative heterochromatin shows similarly depleted bleb to body ratios as DNA, while constitutive heterochromatin has an increased bleb to body ratio relative to DNA.", again interpreting results as K27me and K9me ratios are different overall, while 8/11 shown panels showed insignificant differences.

Response: The reviewer ask for clarification on the importance of statistical significance between DNA, H3K27me3, and H3K9me2,3. The hypothesis stated in the first sentence of this section is: "Decreased chromatin density in the nuclear bleb could arise from loss of heterochromatin." From this hypothesis we draw the true statistical analysis is that both H3K27me3 and H3K9me2,3 should be similarly depleted in the nuclear bleb compared to the body relative to DNA. Our data refute this hypothesis because H3K9me2,3 has an increased nuclear bleb to body ratio relative to DNA, while H3K27me3 does not. Thus, we are not aiming to determine a difference between H327me3 and H3K9me2,3 – just whether both are deleted in their nuclear bleb to body ratio in comparison to DNA. Below we have revised sentences to more strongly focus on this comparison.

- P.2 "Thus, in MEF cells facultative heterochromatin is similar to DNA nuclear bleb to body ratio while constitutive heterochromatin is increased relatively to DNA.
- Fig3A H3K27me and DNA are statistically similar while H3K9me2,3 and DNA are significantly different
- P.3 "Taken together, constitutive heterochromatin is not depleted in the nuclear bleb relative to the nuclear body like DNA while facultative heterochromatin is depleted similar to DNA.
- P. 4 "bleb to body ratio relative to DNA" is clearly worded here. In this sentence we clearly outline the comparisons of DNA and H3K27me3 as well as DNA and H3K9me2,3 which is supported by the data. Thus, no change is needed here
- To further help clarify this point we also changed the section heading to : " Constitutive Heterochromatin shows a differential behavior in nuclear bleb to body ratio relative to DNA."

Fourth decision letter

MS ID#: bio.062585R3

MS Title: Nuclear blebs are composed of variable chromatin states and display increased transcription initiation relative to elongation

Authors: Madeleine E. Clark; Antonela Losada; Sonia E. Jahng; Allen Saini; Fawwaz A. Chowhan; Gwyneth L. Woods; Adelaide S. Cutler; Stephanie A. Hallerman; Minnah A. Gayed; Sayali R. Bhalerao; Emanuel Bullock; Chris S. Santry; Adriana G. Panagiotou; Bailey Lapolla; Nitish Bhatta; Savanah J. Freidus; Gurnoor Kaur; David Bai; Daniel Hu; Kian Tadbiri; Mary Packard; Katherine

© 2026. Published by The Company of Biologists under the terms of the Creative Commons Attribution License (https://creativecommons.org/licenses/by/4.0/).

Dorfman; Nickolas Borowski; Kelsey Prince; Nick Lang; Carline Fermino do Rosário; Andrew D. Stephens

I am happy to tell you that your manuscript has been accepted for publication in Biology Open, pending our standard publication integrity checks. It was accepted on 30th September 2026.

© 2026. Published by The Company of Biologists under the terms of the Creative Commons Attribution License (https://creativecommons.org/licenses/by/4.0/).
